# Early Anesthesia-Period Stress Hyperglycemia Ratio and In-Hospital Mortality After Noncardiac Surgery in Patients with HbA1c and Glucose Measurements

**DOI:** 10.3390/jcm15208002

**Published:** 2026-10-15

**Authors:** Jianhao Zhang, Yuguan Zhang, Lingxin Wei

**Affiliations:** 1 Department of Anesthesiology, Plastic Surgery Hospital, Chinese Academy of Medical Sciences & Peking Union Medical College, No. 33 Badachu Road, Shijingshan District, Beijing 100144, China; zhangjh20001029@163.com; 2 Department of Anesthesiology, Peking Union Medical College Hospital, Chinese Academy of Medical Sciences & Peking Union Medical College, No. 1 Shuaifuyuan, Wangfujing, Dongcheng District, Beijing 100730, China; zygpumch@126.com

**Keywords:** stress hyperglycemia ratio, perioperative hyperglycemia, noncardiac surgery, anesthesia, in-hospital mortality, INSPIRE

## Abstract

**Background/Objectives:** We examined the association between early anesthesia-period relative hyperglycemia and in-hospital mortality after noncardiac surgery. **Methods:** This retrospective INSPIRE cohort included admissions with pre-anesthesia HbA1c and glucose measured within 60 min after anesthesia start. Early iSHR was first glucose divided by HbA1c-derived estimated average glucose; whole-anesthesia peak iSHR was secondary. Follow-up extended from anesthesia end until death or same-admission discharge, with variable duration. Logistic spline and linear models used patient-clustered standard errors. **Results:** Of 117,429 source admissions, 10,406 were included, with 101 deaths. The core-adjusted OR per 0.10 increase in early iSHR was 1.134 (95% CI 1.063–1.210). Further adjustment for measurement count, renal function, malignancy, hypotension, and blood-product records yielded an OR of 1.089 (1.014–1.170) in 10,352 admissions with 97 deaths. For whole-anesthesia peak iSHR (11,374 admissions; 104 deaths), otherwise matched models without and with low-MAP proportion and blood-product records yielded ORs of 1.126 (1.063–1.193) and 1.064 (0.999–1.133; *p* = 0.052), respectively. A separate TWA/count model yielded an OR of 1.039 (0.973–1.109) in 11,312 admissions. Internal comparisons showed no clear discrimination advantage over absolute early glucose. **Conclusions:** In clinically selected patients with pre-anesthesia HbA1c and early glucose measurements, early iSHR was associated with in-hospital mortality in core and further-adjusted models, although creatinine-complete and influence analyses indicated limited robustness. The whole-anesthesia peak-iSHR estimate attenuated and showed no clear association when hypotension and blood-product records were included; the TWA/count model also showed no clear association. Internal comparison did not establish a predictive advantage over absolute early glucose; causality cannot be inferred.

## 1. Introduction

Perioperative glucose elevation reflects the metabolic response to stress [1,2] and is associated with infection, acute kidney injury, and death after noncardiac surgery [3,4,5,6]. However, risk thresholds for absolute glucose differ across studies. A multicenter study found no independent association between peak intraoperative glucose >180 mg/dL and a 30-day composite outcome, whereas risk was higher at glucose ≥250 mg/dL [7]. Another study associated an intraoperative peak glucose of at least 180 mg/dL with myocardial injury in patients with diabetes [8].

The metabolic change represented by an acute glucose concentration depends on the patient’s chronic glycemia. At similar perioperative glucose levels, patients without diabetes may have a higher complication risk than those with diabetes [5,9], potentially reflecting a greater rise relative to their usual glucose level [10]. The stress hyperglycemia ratio (SHR), calculated as acute glucose divided by HbA1c-derived estimated average glucose (eAG), quantifies this relative elevation [11]. Studies in critically ill populations have associated relative hyperglycemia with mortality and reported differences across diagnostic groups [12,13,14].

Previous INSPIRE studies examined preoperative or admission SHR in relation to cardiovascular outcomes [15,16], ICU admission after gastrointestinal tumor surgery [17], and postoperative AKI [18]. These studies did not directly address early anesthesia-period relative hyperglycemia. Interpreting a whole-anesthesia maximum also requires attention to measurement frequency: more measurements increase the opportunity to observe a high value. Clinically determined monitoring frequency may reflect illness severity [19].

We examined the association between first-60 min iSHR and in-hospital mortality after noncardiac surgery, with whole-anesthesia peak iSHR as the secondary exposure. Same-cohort comparisons of iSHR, absolute glucose, and glucose plus eAG assessed their prognostic information. Exploratory analyses examined early iSHR in relation to ICU admission, myocardial infarction or stroke, and postoperative AKI.

## 2. Materials and Methods

### 2.1. Study Design and Data Source

This retrospective cohort study used INSPIRE version 1.4.2 from PhysioNet, containing perioperative data from Seoul National University Hospital, Republic of Korea, during 2011–2020 [20,21,22]. Database release was approved by the hospital Institutional Review Board (H-2210-078-1368), with informed consent waived. Reporting follows STROBE. The original statistical analysis plan designated whole-anesthesia peak iSHR as the primary exposure. After exploratory analyses, a dated amendment designated the first iSHR measured within 60 min of anesthesia start as the primary exposure.

### 2.2. Study Population

Adults undergoing noncardiac surgery were eligible if HbA1c was available within 90 days before anesthesia and glucose was measured at least once during anesthesia. The first operation per admission was retained. The primary analysis additionally required glucose within [anesthesia start, min(anesthesia start + 60 min, anesthesia end)]. Surgical specialties and detailed cohort rules are provided in the Supplementary Methods.

The analysis unit was the surgical admission. Multiple admissions per patient were allowed, with standard errors clustered by patient (Tables S1 and S2).

### 2.3. Exposure Definition

Early iSHR was the first glucose in the early anesthesia window divided by estimated average glucose (eAG), calculated from the most recent eligible HbA1c [23]:

eAG (mg/dL) = 28.7 × HbA1c (%) − 46.7.

Whole-anesthesia peak iSHR was the highest measured glucose between anesthesia start and end divided by the same eAG. Both exposures were modeled continuously; quartiles were used descriptively. Glucose and HbA1c were the anonymized representative values released by INSPIRE. The INSPIRE data descriptor reports that intraoperative laboratory tests were primarily point-of-care arterial blood gas analyses in patients with arterial catheters [21]. The released glucose records do not identify the testing method for individual measurements.

### 2.4. Outcome Definitions

The primary outcome was death after anesthesia end and before discharge from the same admission. This endpoint was chosen because the released data identify in-hospital death and discharge for the index admission, whereas deaths after discharge within 30 days of anesthesia end could not be reliably ascertained. Sensitivity outcomes were in-hospital death within 7 and 30 days after anesthesia end.

Exploratory secondary outcomes were ICU admission, newly recorded myocardial infarction or stroke, and creatinine-defined AKI. ICU admission was identified from the available surgery-anchored records as the first entry within 24 h after anesthesia end and before discharge, excluding patients already in ICU at anesthesia end. The database recording window and risk-set rules are detailed in the Supplementary Methods.

Myocardial infarction or stroke was identified from new I21–I22 or I60–I64 diagnosis records after anesthesia end and before discharge or death; corresponding diagnoses recorded earlier in the admission were excluded.

Using KDIGO creatinine criteria, AKI was defined by a creatinine increase of ≥0.3 mg/dL within 48 h or ≥1.5 times the pre-anesthesia baseline within 7 days after anesthesia end, before discharge [24]. Baseline eligibility, postoperative measurement requirements, and outcome-specific risk sets are detailed in the Supplementary Methods and Table S11.

### 2.5. Covariates

Core adjustment included age, sex, ASA physical status, emergency status, diabetes, HbA1c, anesthesia duration, and surgical specialty. The early-iSHR models also adjusted for time from anesthesia start to the first glucose measurement. Anesthesia duration represented procedure length and glucose-monitoring opportunity. The further-adjusted early-iSHR model added whole-anesthesia glucose count, baseline creatinine and its missingness indicator, malignancy, blood-product records, and time-weighted mean arterial pressure deficit below 65 mmHg (TWA65). For whole-anesthesia peak iSHR, two models were compared in the same 11,374 admissions after common adjustment for core covariates, creatinine, and malignancy. One excluded hypotension and blood-product terms if these were considered possible events after the glycemic response; the other included low-MAP reading proportion and blood-product records if these mainly reflected shared operative severity. Graded TWA65/TWA55 and measurement-count analyses were evaluated separately. Covariate coding and exposure-specific model specifications are provided in the Supplementary Methods.

### 2.6. Statistical Analysis

Continuous variables are summarized as mean (standard deviation) or median (interquartile range), and categorical variables as number (percentage). Standardized mean differences compared mutually exclusive included and nonincluded admissions and early- versus later-measured admissions.

The primary analysis used logistic regression with patient-clustered robust standard errors and a three-knot restricted cubic spline at the 10th, 50th, and 90th exposure percentiles, referenced to iSHR = 1.00. Wald tests assessed overall association and nonlinearity. A separate linear model reported the OR per 0.10 increase. Model-standardized mortality risks were averaged over the cohort’s covariate distribution.

Early and peak iSHR were compared within identical admissions and adjustment sets. A joint glucose/eAG model examined their mutually adjusted associations with mortality, omitting the separate HbA1c term. Internal performance of absolute first glucose, early iSHR, and first glucose plus eAG was compared using common clinical covariates and 1000 paired patient-level bootstrap samples [25].

Sensitivity analyses addressed hypotension definitions, creatinine availability, sparse events using Firth regression [26], HbA1c reliability, and influential observations. Exploratory timing analyses examined early-iSHR associations by whole-anesthesia measurement-count category (1, 2, or ≥3); subsequent glucose change was modeled conditional on the first value. For peak iSHR, full-cohort adjustment for glucose-measurement count was the principal exploratory secondary monitoring analysis; restriction to at least two measurements with count adjustment was a separate sensitivity analysis. Detailed specifications are provided in the Supplementary Methods.

In the first-eligible-admission subset, 7- and 30-day in-hospital mortality were analyzed with live discharge as a competing event; 30-day associations used core-adjusted cause-specific Cox and Fine–Gray models. Secondary outcomes used outcome-specific risk sets, linear iSHR for ICU admission, and spline models with separate linear summaries for AKI and myocardial infarction or stroke.

Missing ASA status was coded separately; missing creatinine was median-replaced with a missingness indicator. The primary test was the overall early-iSHR–mortality association (two-sided *p* < 0.05). Secondary and exploratory analyses were not intended for confirmatory inference; their *p* values were unadjusted for multiplicity. Statistical analysis and numerical verification used R 4.3.0 and 4.6.1 (R Foundation for Statistical Computing, Vienna, Austria) and Python 3.12.14 (Python Software Foundation, Beaverton, OR, USA). Implementation details and software versions are provided in the Supplementary Methods.

## 3. Results

### 3.1. Cohort Characteristics

**Figure 1:**
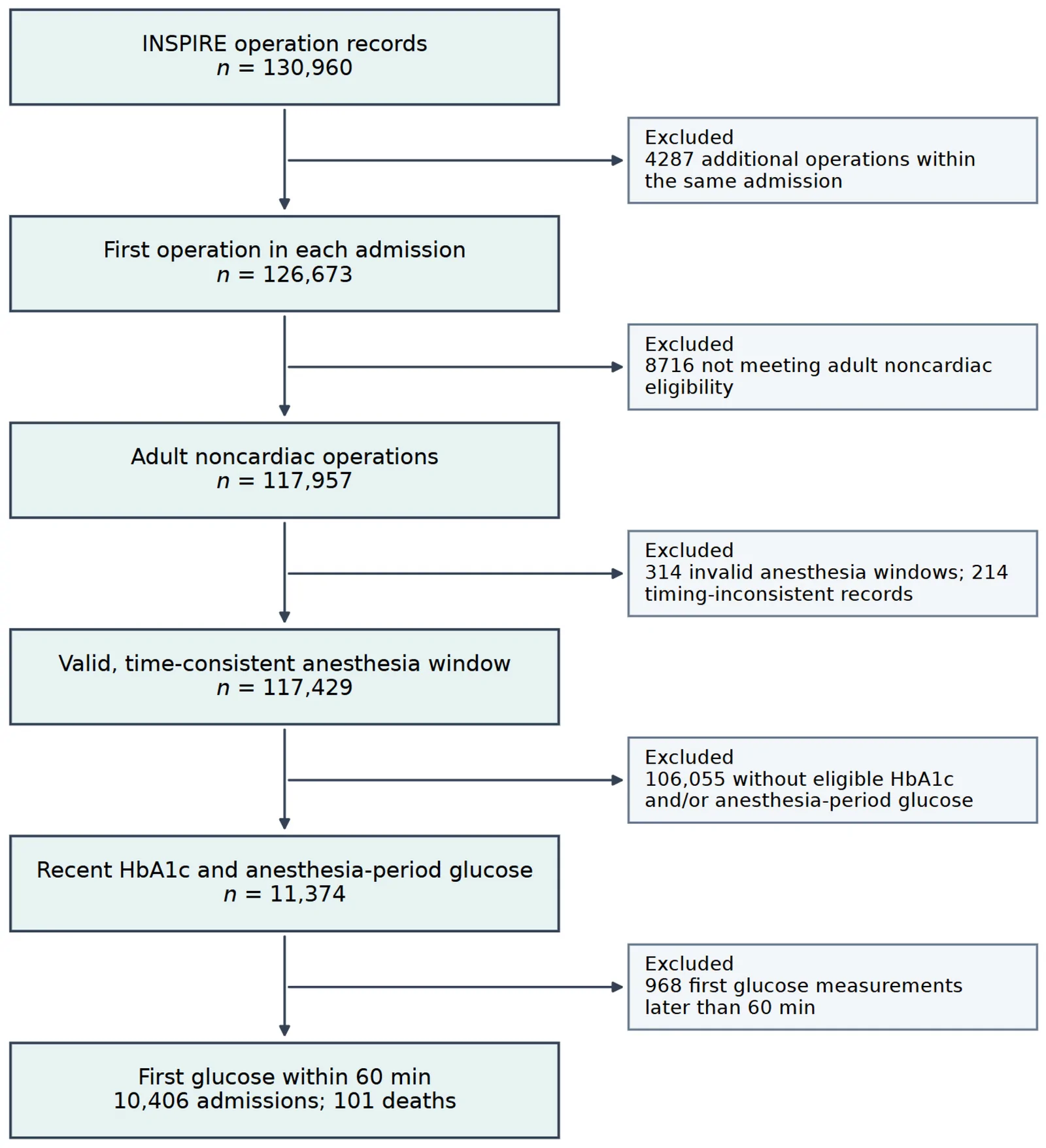
Selection of the peak-iSHR cohort and the first-60 min iSHR cohort.

Among 130,960 surgical records, 117,429 admissions formed the eligible source population. HbA1c and anesthesia-period glucose were available for 11,374 admissions; 10,406 had an early measurement, representing 9888 patients and 101 deaths (Figure 1). The median first-sample time was 25 min after anesthesia start (IQR 20–40; Tables S1 and S2). One source admission had death recorded at or before anesthesia end and did not enter either exposure cohort.

The early-iSHR cohort represented 8.86% of the source population. Compared with nonincluded admissions, included admissions involved older patients, a higher prevalence of diabetes and ASA ≥III, and longer anesthesia; the absolute SMD for log2 anesthesia duration was 1.021 (Table 1, Panel B). The whole-anesthesia peak-iSHR cohort showed the same selection direction (Table 1, Panel C). Within the peak cohort, admissions first measured after 60 min had longer anesthesia than early-measured admissions (median 250 versus 195 min; Table S2).

In both the early and full peak-iSHR cohorts, whole-anesthesia glucose count was higher among admissions ending in death than among survivors: median 3 (IQR 2–5) versus 2 (1–3). The median was 2 (1–3) in each early-iSHR quartile. In the full peak-iSHR cohort, median counts across peak-iSHR quartiles Q1–Q4 were 1 (IQR 1–2), 2 (1–3), 2 (2–3), and 3 (2–4), respectively. Whole-anesthesia counts and early-window counts are reported separately in Table S3 and Figure S1.

The peak-iSHR cohort included 11,374 admissions. Exclusion of 968 admissions with the first anesthesia-period glucose measurement after 60 min yielded the early-iSHR primary cohort. For anesthesia lasting less than 60 min, the sampling window ended at anesthesia completion. Counts are at the admission level.

### 3.2. Early Relative Hyperglycemia and In-Hospital Mortality

**Figure 2:**
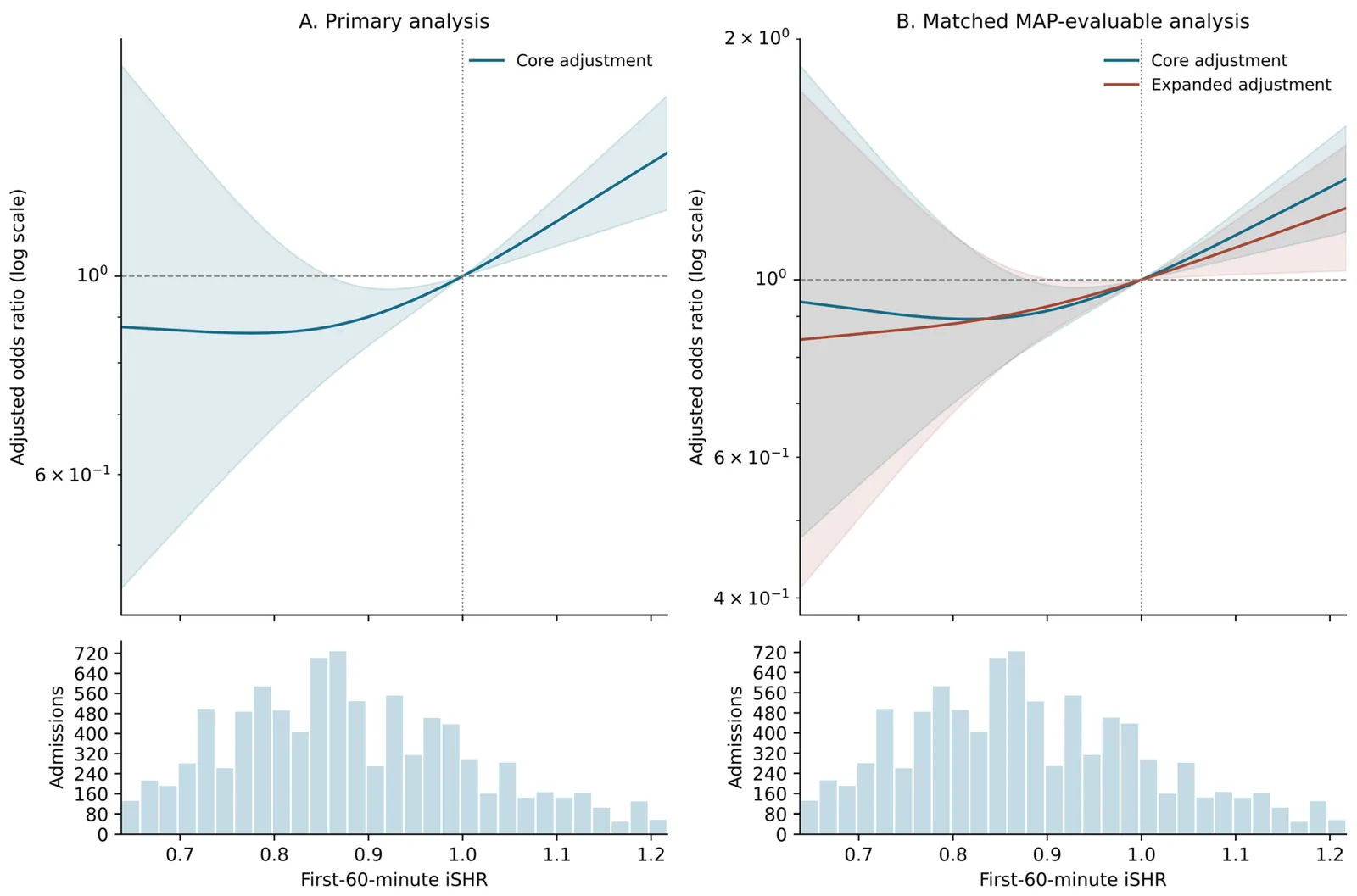
First-60 min iSHR and in-hospital mortality under core and expanded adjustment.

First-60 min iSHR was associated with in-hospital mortality in the core model (overall RCS *p* = 5.97 × 10^−5^); the nonlinearity test gave *p* = 0.304. The OR was 1.339 (1.170–1.533) for 1.20 versus 1.00 and 2.104 (1.481–2.989) for 1.50 versus 1.00. The separate linear estimate was 1.134 (1.063–1.210) per 0.10. Model-standardized risks were 8.88 and 17.80 per 1000 at 1.00 and 1.50, respectively (Table 2; Figure 2; Table S10).

In the MAP-evaluable subset (10,352 admissions; 97 deaths), expanded adjustment attenuated the linear OR from 1.118 (1.046–1.195) to 1.089 (1.014–1.170). The expanded spline overall *p* value was 0.047 (nonlinearity *p* = 0.689; Figure 2). Estimates under alternative hypotension definitions are provided in Table S4.

Core models adjust for clinical covariates and the interval from anesthesia start to the first glucose measurement. Expanded models additionally adjust for measurement count, creatinine and its missingness indicator, malignancy, blood-product records, and TWA65. RCS contrasts use iSHR = 1.00 as the reference; linear ORs are per 0.10, with patient-clustered 95% CIs. Standardized risks are estimated from the core RCS model. There were 153 admissions with iSHR ≥ 1.50; this value is within the observed range but above the central range displayed in Figure 2. Sensitivity analyses are in Tables S4 and S5.

Panel A uses 10,406 admissions and 101 deaths. Panel B overlays the core and expanded models in the identical MAP-evaluable subset of 10,352 admissions and 97 deaths. Solid lines are adjusted ORs, shaded bands 95% patient-clustered CIs, and the reference is 1.00. The OR axis is logarithmic. Histograms show the distribution of first-60 min iSHR in each panel’s analysis cohort. The display spans 0.6376–1.2173 (5th–95th percentiles): 9353 admissions in Panel A and 9311 in Panel B are inside, with 1053 and 1041 outside. All eligible observations, including those outside the display, were used for fitting. Knots were 0.69410913, 0.86776860, 1.12151356.

### 3.3. Timing, Glycemic Background, and Internal Performance

Early and peak iSHR were identical in 4748 admissions (45.6%). Their same-cohort core-adjusted associations were similar, with greater attenuation for peak iSHR after measurement-count adjustment (Table S4). In the full peak-iSHR cohort (11,374 admissions; 104 deaths), adding log2 measurement count to the core model yielded an OR of 1.086 (1.022–1.154) per 0.10 increase in peak iSHR (Table S16). The early-iSHR association did not clearly differ across whole-anesthesia count categories (interaction *p* = 0.441; Table S14). Conditional on first glucose, subsequent rise was not clearly associated with mortality, either before or after count adjustment (Table S15). In the whole-anesthesia peak-iSHR core model, adjusted ORs were 1.00 (95% CI 0.80–1.25) for HbA1c per 1% increase and 1.37 (0.83–2.25) for preoperative diabetes. In the whole-anesthesia peak-glucose/eAG joint model, the equal-and-opposite coefficient restriction was not rejected (Wald *p* = 0.845; *p* = 0.791 after glucose-count adjustment; Table S18). On the modeled concentration scale, this is a glucose–eAG difference restriction.

**Figure 3:**
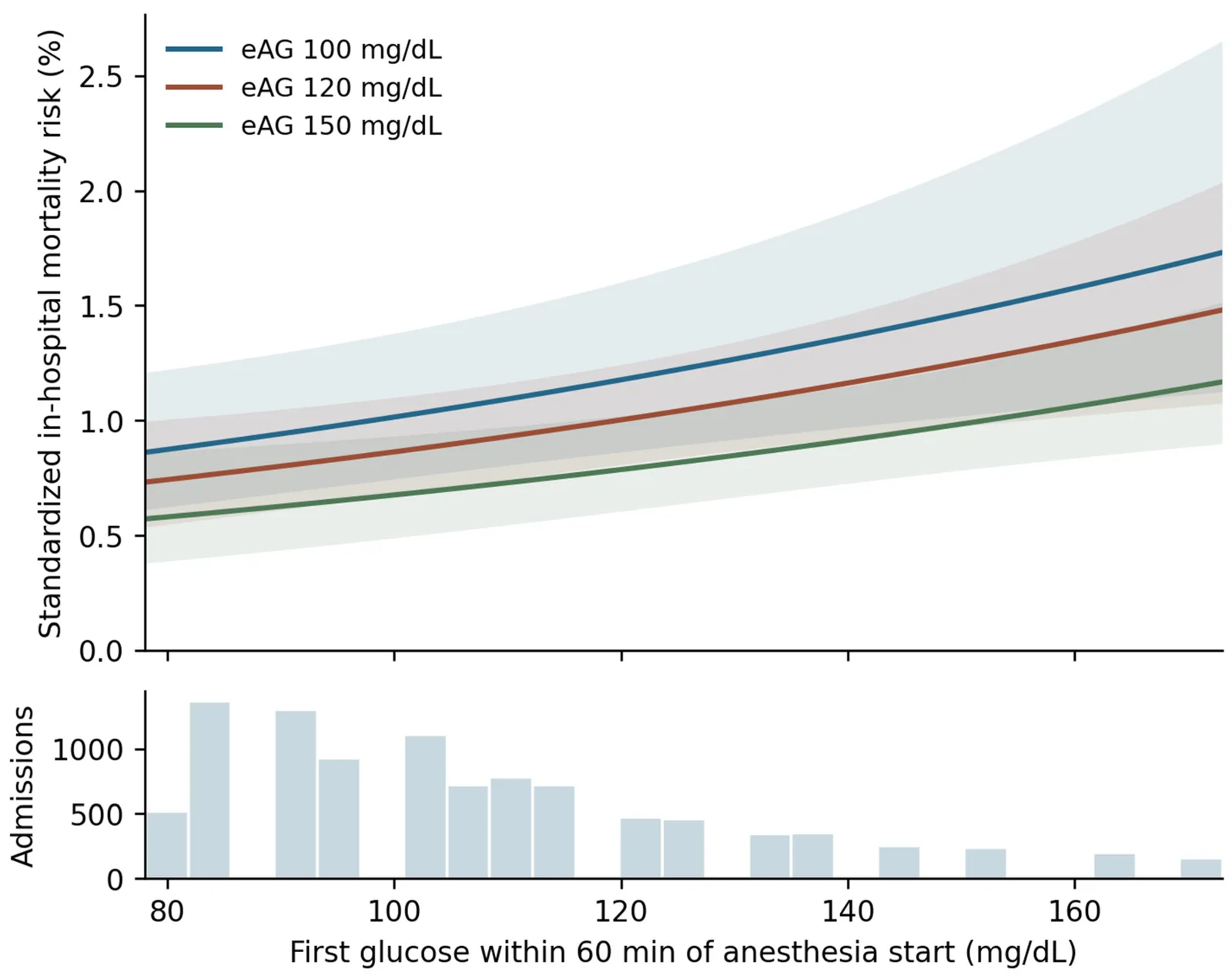
Early glucose and chronic glycemia in relation to standardized mortality risk.

In the joint model, the ORs per 20 mg/dL were 1.173 (1.058–1.301) for first glucose and 0.841 (0.708–0.999) for eAG. Thus, the estimated mortality risk associated with a given first glucose differed by chronic glycemic level (Figure 3; Table S13).

Curves show mortality risks from the joint first-glucose/eAG model in 10,406 admissions with 101 deaths, standardized over the observed common-covariate distribution. The adjustment set comprises age, sex, ASA, emergency status, diabetes, log2 anesthesia duration, surgical specialty, first-sample timing, and log2 whole-anesthesia glucose count, without a separate HbA1c term. eAG is fixed at 100, 120, or 150 mg/dL; shaded bands indicate pointwise 95% patient-clustered delta-method CIs. The model contains no glucose-by-eAG interaction. The display spans the 5th–95th percentiles of first glucose (78–173 mg/dL), containing 9988 admissions; 418 lie outside, and all 10,406 contributed to fitting. The lower panel shows the first-glucose distribution. These are model-standardized risks, not observed rates within eAG subgroups. eAG denotes HbA1c-derived estimated average glucose.

The optimism-corrected C-statistics for absolute first glucose, early iSHR, and first glucose plus eAG were 0.8640, 0.8664, and 0.8664, respectively. The paired apparent C-statistic difference for early iSHR versus absolute glucose was 0.0025 (95% CI −0.0013–0.0069). Calibration and Brier scores are reported in Table 3 and Table S7, with apparent calibration in Figure S3.

The optimism-corrected C-statistics for absolute peak glucose, peak iSHR, and peak glucose plus eAG were 0.8654, 0.8674, and 0.8673, respectively (Table 3, Panel C). The paired apparent C-statistic difference for peak iSHR versus absolute peak glucose was 0.0019 (95% CI −0.0018–0.0059; Table S18).

In Panels A–B, the three early glycemic models include 10,406 admissions, 9888 patients, and 101 deaths, with the same clinical adjustment: core covariates excluding a separate HbA1c term, plus total glucose count. Nonintercept parameters are 13, 13, and 14. Corrected point estimates subtract mean bootstrap optimism; confidence intervals describe refitted apparent performance. Each model had 1000 valid paired repetitions. Calibration results are provided in Table S7.

Panel C uses the distinct whole-anesthesia peak cohort (11,374 admissions, 10,782 patients, and 104 deaths), whereas Panels A–B use the early cohort (10,406 admissions, 9888 patients, and 101 deaths). All three Panel C models use the same comparison covariates: core covariates excluding a separate HbA1c term plus log2 whole-anesthesia glucose count. Apparent C 95% CIs are bootstrap percentile intervals, not uncertainty intervals for optimism-corrected point estimates; paired-difference CIs refer only to apparent differences. Paired differences and coefficient-sum restrictions are reported in Table S18.

### 3.4. Sensitivity Analyses and Model Evaluation

The primary early-iSHR analysis included 10,406 admissions and 101 deaths (Table 2). In separate first-eligible-admission sensitivity subsets, Firth estimates were close to matched maximum-likelihood estimates (Table S5). In the creatinine-complete MAP subset (6505 admissions; 88 deaths), the core and expanded ORs per 0.10 were 1.105 (1.029–1.186) and 1.071 (0.994–1.154), respectively (Table S5). HbA1c-reliability sensitivity analyses are presented in Table S8.

The median interval from anesthesia end to death or discharge was 6.5 days (IQR 4.4–10.3); 38 of 101 deaths occurred after day 30. In the first-admission early subset, 22 and 56 deaths occurred within 7 and 30 days, respectively. The core-adjusted 30-day cause-specific and subdistribution HRs per 0.10 increase were 1.159 (1.074–1.250) and 1.196 (1.104–1.296), respectively (Table S6; Figure S2). In the whole-anesthesia peak-iSHR first-admission subset (10,782 admissions; 58 deaths by day 30), the corresponding 30-day in-hospital cause-specific HR was 1.122 (95% CI 1.047–1.201) per 0.10 (Table S6 note).

In influence analyses, the expanded spline OR for early iSHR 1.50 versus 1.00 decreased from 1.617 to as low as 1.415 (95% CI 0.908–2.207), indicating sensitivity to individual observations (Table S9). Internal calibration is shown in Figure S4.

In 11,374 admissions with 104 deaths, the whole-anesthesia peak-iSHR OR per 0.10 was 1.126 (1.063–1.193) after adjustment for core covariates, creatinine, and malignancy, and 1.064 (0.999–1.133) when low-MAP reading proportion and blood-product records were also included (Table S17). The peak-iSHR association curve and observed distribution are shown in Figure S6. In the separate TWA/count-eligible subset of 11,312 admissions with 100 deaths, the OR was 1.039 (0.973–1.109; Table S17). The repeat-measurement sensitivity analysis included 7948 admissions with at least two glucose measurements and 79 deaths (Table S16).

### 3.5. Exploratory Secondary Outcomes

Early iSHR was positively associated with ICU admission after measurement-count adjustment (OR 1.130 [1.092–1.169] per 0.10). The AKI spline analysis showed evidence of nonlinearity (*p* = 0.046). For myocardial infarction or stroke, the overall spline *p* value was 0.017, while the OR for 1.50 versus 1.00 was 1.453 (0.923–2.289). Outcome-specific populations and estimates are presented in Table 4, Tables S11 and S12, and Figure S5.

All models use early iSHR and patient-clustered 95% CIs. Core covariates include age, sex, ASA, emergency status, diabetes, HbA1c, log2 anesthesia duration, surgical specialty, and first-sample timing. Panel A uses knots 0.69410913, 0.86776860, and 1.12151356 and reference 1.00; Panel B uses linear iSHR, with count adjustment as log2(count/2). Outcome-specific eligibility, all linear estimates, and diagnostics are in Tables S11 and S12. *p* values are unadjusted exploratory tests.

## 4. Discussion

Higher early iSHR was associated with in-hospital mortality after noncardiac surgery, with attenuation after additional adjustment for measurement count, renal function, and anesthesia-period factors. Creatinine-complete and influence analyses indicated limited robustness, while peak iSHR showed no clear association after expanded adjustment. These findings place the prognostic interpretation of anesthesia-period relative hyperglycemia in the context of clinical status and monitoring. Peak iSHR is additionally influenced by monitoring intensity because more frequent testing increases the opportunity to observe a high value. In the whole-anesthesia peak-iSHR analysis, longer anesthesia was associated with entry into the glucose-tested cohort and also increased opportunities to observe a high whole-anesthesia peak. The inverse, imprecise duration estimate (OR 0.77 per doubling; 95% CI 0.55–1.07; Table S17) may reflect short emergency or otherwise urgent procedures and cases requiring postoperative ICU care. Although emergency status and ASA were included in adjustment, surgical specialty and anesthesia duration cannot fully represent procedural risk.

The first early glucose was also the whole-anesthesia maximum in nearly half of admissions. The median sampling interval was 25 min after anesthesia start, and subsequent rise showed no clear mortality association after adjustment for the first value. Thus, in many admissions the whole-anesthesia peak reflected relative hyperglycemia already present early, while its observed height also depended on measurement opportunities. Prognostic information was therefore observable early in anesthesia, although the measurement also reflected preoperative illness, anesthesia and treatment, and observed glucose changes depended on sampling timing and frequency [19].

The attenuation of the peak-iSHR estimate after adding hypotension and blood-product records has two plausible interpretations. If these variables reflect operative severity shared by the glycemic response and mortality, their inclusion addresses part of that confounding. If they occur after glycemic stress, including them may remove part of the broader association. Both variables were summarized over the full anesthesia period; the models did not establish their order relative to the observed glucose peak or estimate mediation. The median interval from anesthesia end to death was 20.4 days; subsequent illness and treatment may also contribute to mortality. Peak iSHR is therefore interpreted as a context-dependent prognostic marker of perioperative risk.

The early-glucose/eAG joint model estimated different mortality risks at the same first glucose across chronic glycemic levels, supporting interpretation of acute glucose in its chronic glycemic context. However, early iSHR and absolute early glucose had similar discrimination, without a clear predictive advantage for the ratio.

Previous INSPIRE studies mainly examined preoperative or admission SHR in relation to cardiovascular events, ICU admission, and AKI [15,16,17,18] (Table 5). Individual overlap is unquantifiable from published aggregate data. Our analyses address anesthesia-period glycemia, comparing early first values with whole-period peaks in the same admissions and examining the influence of clinical monitoring and perioperative factors.

Early iSHR was also associated with ICU admission, the AKI association was nonlinear, and the high-value contrast for myocardial infarction or stroke was imprecise. These findings extend the observations to other postoperative outcomes, although ICU admission may include planned monitoring and AKI assessment depends on postoperative creatinine testing. Confirmation in cohorts with more complete measurements and follow-up is needed.

The main limitations are selective clinical testing, residual confounding, and few deaths. Selective HbA1c and early glucose testing at a single center limits the applicability of these findings to the broader noncardiac surgical population. The included group’s higher diabetes and ASA ≥III prevalence, older age, and longer anesthesia are consistent with HbA1c testing for known or suspected dysglycemia and glucose monitoring when greater perioperative surveillance is anticipated. The released five-character procedure codes [20] did not support reliable surgical-severity classification using the SORT framework [27], and insulin, corticosteroid, and prior transfusion data were incomplete. Representative-value anonymization and unidentified assay sources add measurement uncertainty. Follow-up was confined to the same admission; ICU ascertainment depended on the database recording window, and AKI was assessed using creatinine criteria.

Prospective multicenter studies could standardize early glucose measurement times, improve assessment of chronic glycemia, procedural risk, and treatment, and include post-discharge follow-up. Such data would support further evaluation of early iSHR for perioperative risk assessment.

## 5. Conclusions

Among clinically selected noncardiac surgical patients with pre-anesthesia HbA1c and early glucose measurements, higher early iSHR was associated with in-hospital mortality. Expanded adjustment reduced the estimated effect, and creatinine-complete and influence analyses indicated limited robustness. Early iSHR may be considered a context-dependent prognostic marker; internal comparison did not show a clear discrimination advantage over absolute early glucose.

## Figures and Tables

**Table 1:** Characteristics and selection of early and whole-anesthesia peak-iSHR cohorts.

Panel A. Early-evaluable admissions by first-60 min iSHR quartile					
**Characteristic**	**Overall**	**Q1**	**Q2**	**Q3**	**Q4**
Admissions	10,406	2622	2765	2459	2560
Age, years; mean (SD)	59.5 (14.7)	61.9 (13.0)	57.9 (15.2)	59.0 (15.2)	59.2 (14.8)
Male, *n* (%)	4953 (47.6)	1206 (46.0)	1234 (44.6)	1183 (48.1)	1330 (52.0)
ASA ≥ III, *n* (%)	1651 (15.9)	529 (20.2)	350 (12.7)	297 (12.1)	475 (18.6)
ASA missing, *n* (%)	174 (1.7)	45 (1.7)	36 (1.3)	35 (1.4)	58 (2.3)
Emergency, *n* (%)	763 (7.3)	207 (7.9)	162 (5.9)	146 (5.9)	248 (9.7)
Preoperative diabetes, *n* (%)	1741 (16.7)	658 (25.1)	315 (11.4)	313 (12.7)	455 (17.8)
Anesthesia duration, min	195.0 (135.0–285.0)	195.0 (135.0–290.0)	190.0 (135.0–280.0)	195.0 (140.0–282.5)	205.0 (140.0–295.0)
HbA1c, %	5.8 (5.3–6.4)	6.3 (5.8–7.1)	5.7 (5.3–6.1)	5.7 (5.3–6.1)	5.4 (5.2–6.1)
First glucose within 60 min, mg/dL	101.0 (91.0–120.0)	91.0 (84.0–105.0)	96.0 (91.0–105.0)	105.0 (96.0–120.0)	126.0 (110.0–153.0)
First-60 min iSHR	0.868 (0.776–0.984)	0.709 (0.654–0.741)	0.824 (0.798–0.857)	0.922 (0.896–0.956)	1.085 (1.024–1.188)
Time to first glucose, min	25.0 (20.0–40.0)	25.0 (20.0–35.0)	25.0 (15.0–35.0)	25.0 (20.0–35.0)	30.0 (20.0–40.0)
Whole-anesthesia glucose count	2.0 (1.0–3.0)	2.0 (1.0–3.0)	2.0 (1.0–3.0)	2.0 (1.0–3.0)	2.0 (1.0–3.0)
Creatinine available, *n* (%)	6537 (62.8)	1608 (61.3)	1573 (56.9)	1568 (63.8)	1788 (69.8)
General surgery, *n* (%)	2586 (24.9)	693 (26.4)	643 (23.3)	600 (24.4)	650 (25.4)
Orthopedic surgery, *n* (%)	2216 (21.3)	440 (16.8)	532 (19.2)	618 (25.1)	626 (24.5)
Neurosurgery, *n* (%)	1584 (15.2)	385 (14.7)	345 (12.5)	286 (11.6)	568 (22.2)
Other specialty, *n* (%)	4020 (38.6)	1104 (42.1)	1245 (45.0)	955 (38.8)	716 (28.0)
In-hospital deaths, *n* (%)	101 (1.0)	22 (0.8)	14 (0.5)	19 (0.8)	46 (1.8)
Panel B. Mutually exclusive included and nonincluded source admissions					
**Characteristic**	**Included (*n* = 10,406)**		**Not Included (*n* = 107,023)**		**SMD**
Age, years; mean (SD)	59.5 (14.7)		54.9 (16.2)		0.296
Male	4953 (47.6)		45,575 (42.6)		0.101
ASA ≥ III	1651 (15.9)		6924 (6.5)		0.302
ASA missing	174 (1.7)		2604 (2.4)		−0.054
Emergency	763 (7.3)		8744 (8.2)		−0.031
Preoperative diabetes	1741 (16.7)		7241 (6.8)		0.313
Anesthesia duration, min	195.0 (135.0–285.0)		105.0 (65.0–170.0)		1.021
General surgery	2586 (24.9)		31,393 (29.3)		−0.101
Orthopedic surgery	2216 (21.3)		13,678 (12.8)		0.228
Neurosurgery	1584 (15.2)		7819 (7.3)		0.252
Other specialty	4020 (38.6)		54,133 (50.6)		−0.242
Panel C. Whole-anesthesia peak-iSHR cohort versus mutually exclusive nonincluded source admissions					
**Characteristic**	**Included (*n* = 11,374)**		**Not Included (*n* = 106,055)**		**SMD**
Age, years; mean (SD)	59.4 (14.6)		54.9 (16.2)		0.292
ASA ≥ III, *n* (%)	1789 (15.7)		6786 (6.4)		0.301
Preoperative diabetes, *n* (%)	1934 (17.0)		7048 (6.6)		0.325
Anesthesia duration, min; median (IQR)	200 (140–290)		105 (65–170)		1.063
Neurosurgery, *n* (%)	1759 (15.5)		7644 (7.2)		0.263

Panel A: Values are mean (SD), median (IQR), or *n* (%). Quartile cut points were 0.7758, 0.8678, and 0.9838, with tied values assigned to the same category. HbA1c, glycated hemoglobin; ASA, American Society of Anesthesiologists; iSHR, anesthesia-period stress hyperglycemia ratio. Panel B: Values are mean (SD), median (IQR), or *n* (%). Included and nonincluded admissions are mutually exclusive. SMDs use included minus nonincluded, with anesthesia duration on the log2 scale. ASA missingness is shown separately. Panel C compares mutually exclusive groups; anesthesia-duration SMD uses log2 duration.

**Table 2:** Primary mortality analysis and matched expanded analyses of first-60 min iSHR.

Analysis/Contrast	Admissions	Deaths	Estimate (95% CI)	*p* Value
Early iSHR: core, linear	10,406	101	1.134 (1.063–1.210) per 0.10	1.30 × 10^−4^
Early iSHR: core, RCS	10,406	101	Overall/nonlinear Wald	5.97 × 10^−5^/0.304
Same MAP subset: core, linear	10,352	97	1.118 (1.046–1.195) per 0.10	0.001
TWA65 + count expanded, linear	10,352	97	1.089 (1.014–1.170) per 0.10	0.019
TWA65 + count expanded, RCS	10,352	97	Overall/nonlinear Wald	0.047/0.689
Core RCS: 1.20 vs. 1.00	10,406	101	1.339 (1.170–1.533)	2.23 × 10^−5^
Core RCS: 1.50 vs. 1.00	10,406	101	2.104 (1.481–2.989)	3.28 × 10^−5^
Expanded RCS: 1.20 vs. 1.00	10,352	97	1.209 (1.024–1.427)	0.025
Expanded RCS: 1.50 vs. 1.00	10,352	97	1.617 (1.051–2.488)	0.029
Core standardized risk at 1.00	10,406	101	8.88 (6.26–11.49) per 1000	—
Core standardized risk at 1.20	10,406	101	11.70 (8.53–14.88) per 1000	—
Core standardized risk at 1.50	10,406	101	17.80 (11.85–23.75) per 1000	—

**Table 3:** Internal performance of early and whole-anesthesia glycemic expressions.

Panel A. Discrimination and fit					
**Early Glycemic Expression**		**Apparent C (95% CI)**		**Corrected C**	**AIC/BIC**
Absolute first glucose		0.8768 (0.8467–0.9142)		0.8640	906.90/1008.40
First-60 min iSHR		0.8793 (0.8515–0.9165)		0.8664	901.53/1003.03
First glucose + eAG		0.8802 (0.8541–0.9177)		0.8664	904.73/1013.49
Panel B. Optimism-corrected error and calibration					
**Early Glycemic Expression**		**Corrected Brier**		**Corrected Calibration Intercept**	**Corrected Calibration Slope**
Absolute first glucose		0.009205		−0.0051	0.9438
First-60 min iSHR		0.009191		−0.0047	0.9452
First glucose + eAG		0.009223		−0.0053	0.9410
Panel C. Whole-anesthesia peak glycemic expressions					
**Whole-Anesthesia Peak Expression**	**Apparent C (95% CI)**	**Optimism-Corrected C**	**Corrected Brier**	**Corrected Calibration Slope**	**AIC/BIC**
Absolute peak glucose	0.8766 (0.8442–0.9134)	0.8654	0.008736	0.9511	945.50/1040.90
Peak iSHR	0.8785 (0.8468–0.9139)	0.8674	0.008736	0.9522	941.98/1037.39
Peak glucose + eAG	0.8793 (0.8483–0.9146)	0.8673	0.008754	0.9483	944.25/1046.99

**Table 4:** Exploratory secondary outcomes of early iSHR.

Panel A. Early-iSHR restricted cubic-spline models					
**Outcome**	**Admissions**	**Events**	**OR, 1.50 vs. 1.00 (95% CI)**	**Overall *p***	**Nonlinearity *p***
Newly recorded MI or stroke	10,406	100	1.453 (0.923–2.289)	0.017	0.402
Creatinine-defined AKI	5759	394	1.605 (1.150–2.238)	0.021	0.046
Panel B. Early-iSHR ICU-record models (linear OR per 0.10)					
**ICU Linear Model**	**Admissions**	**Events**	**OR per 0.10 (95% CI)**		***p Value***
Core covariates	10,216	1882	1.139 (1.102–1.177)		1.17 × 10^−14^
Core covariates + glucose measurement count	10,216	1882	1.130 (1.092–1.169)		2.48 × 10^−12^

**Table 5:** Related INSPIRE glycemic studies.

Study	Cohort/Exposure Window	Outcome	Distinction
Lyu et al., 2024 [15]	INSPIRE v1.2; 12,899; Admission/preoperative SHR	30- and 90-day MACCE	Different exposure window and outcome
Lin et al., 2024 [16]	INSPIRE v1.1; 5609; Admission SHR	30-day MACE	Different exposure window and outcome
Lin et al., 2026 [17]	INSPIRE; 2102 GI tumor surgeries; Preoperative SHR	Postoperative ICU admission	Restricted surgery group; preoperative exposure
Huang et al., 2026 [18]	INSPIRE v1.2; nested case–control: 269 AKI cases and 1003 controls; Preoperative/admission SHR plus hs-CRP	Postoperative AKI	Different design and exposure
Present study	INSPIRE v1.4.2; 10,406 early-iSHR admissions (primary); 11,374 peak-iSHR admissions (secondary)	Same-admission mortality	Early and peak anesthesia-period exposures; measurement-frequency analyses

All studies use INSPIRE, with differences in study populations, exposure windows, and outcomes.

## Data Availability

The data presented in this study were derived from INSPIRE version 1.4.2, available through PhysioNet at https://doi.org/10.13026/1eay-yc85 under its credentialing and data-use requirements. Patient-level data cannot be redistributed by the authors. Derived analysis data and code are available from the corresponding author on reasonable request, subject to the database license.

## Supplementary Materials

The following supporting information can be downloaded at: https://www.mdpi.com/article/10.3390/jcm15208002/s1 [21,23,24,25,26].
