# Supplementary material for: Early Anesthesia-Period Stress Hyperglycemia Ratio and In-Hospital Mortality After Noncardiac Surgery in Patients with HbA1c and Glucose Measurements
Source: J Clin Med. 2026 Oct 15;15(20):8002. doi: 10.3390/jcm15208002 (PMC13706281; doi:10.3390/jcm15208002)
Supplement: Supplementary file 1 [file jcm-15-08002-s001.zip › Original SAP.pdf]

Statistical Analysis Plan

Peak Anesthesia-Period Stress Hyperglycemia Ratio and Same-Admission Mortality Among Noncardiac Surgical Admissions With Recent HbA1c and Anesthesia-Period Glucose Measurements

1. Purpose

This document records the analysis specifications used for the INSPIRE secondary database study and distinguishes analyses defined before the final model run from those added after outcome inspection.

The analysis specifications were finalized after feasibility and preliminary data review but before the final model run. The plan was not prospectively registered; analyses added subsequently are identified as post hoc.

2. Study population

Adults undergoing noncardiac surgery in INSPIRE version 1.4.2 were eligible. The first operation per admission was retained. Records with invalid or materially discordant anesthesia and surgical times were excluded. The primary analysis required HbA1c within 90 days before anesthesia and at least one glucose measurement from anesthesia start to anesthesia end. The surgical admission was the analysis unit; standard errors were clustered by patient.

3. Primary exposure and outcome

Estimated average glucose (eAG) was calculated as 28.7×HbA1c−46.7 mg/dL. Peak iSHR was the highest glucose measured between anesthesia start and end divided by eAG. The primary outcome was death after anesthesia end and before discharge from the same admission.

4. Primary model defined before the final model run

The primary model used multivariable logistic regression with patient-clustered robust standard errors. Peak iSHR was fitted with a three-knot restricted cubic spline using the 10th, 50th, and 90th percentiles. Overall and nonlinearity Wald tests were reported. Peak iSHR 1.00 was the reference; ORs were estimated at 1.20 and 1.50. A separate linear model reported the OR per 0.10 increase.

The adjustment set comprised age, sex, ASA physical status, emergency status, preoperative diabetes, HbA1c, log2 anesthesia duration, and surgical specialty. Model-standardized mortality risks were estimated at peak iSHR 1.00, 1.20, and 1.50.

5. Analyses specified before the final model run

Selection weighting was to support inference only if weight behavior, effective sample size, and covariate balance met predefined criteria; otherwise only diagnostics would be shown. Robustness analyses retained the first eligible admission per patient and separately required at least two glucose measurements with adjustment for log2 measurement count. Absolute peak glucose, joint peak glucose/eAG models, and glucose-gap parameterizations were specified to clarify exposure interpretation, not to prove predictive superiority.

6. Later secondary and exploratory analyses

First ICU admission after anesthesia, incident myocardial infarction or stroke, creatinine-defined postoperative AKI, the first glucose gap within 60 min, subsequent glucose change, and time-weighted positive glucose gap were developed after the core model and are designated secondary or exploratory. No multiplicity correction was applied.

7. Post hoc analyses

Post hoc analyses examined glucose sampling frequency and timing by mortality status and peak iSHR quartile; full-cohort adjustment for log2 measurement count; first iSHR within 60 min as a fixed early-window exposure; collinearity diagnostics for peak iSHR and HbA1c; expanded confounder models including baseline creatinine, malignancy, intraoperative hypotension, and transfusion; and a cause-specific Cox analysis using the first eligible admission per patient with discharge alive censored.

These analyses are explicitly labeled post hoc in the manuscript. The cause-specific analysis was repeated with administrative censoring at 30 days. Steroid and insulin records were audited but were too sparse for stable adjustment. A validated procedural-complexity score and operative calendar year were unavailable; anesthesia duration and surgical specialty were retained as limited procedural descriptors.

8. Missing data and inference

No imputation was performed for the exposure-defining HbA1c or anesthesia-period glucose values. Missing ASA status was retained as a category. In the expanded baseline-creatinine model, creatinine was median-imputed with a missingness indicator. All tests were two-sided. The primary inference concerned the overall peak iSHR–same-admission mortality association. Secondary and exploratory P values were not adjusted for multiple comparisons.

9. Interpretation

All estimates are associational. The study does not establish a causal effect of hyperglycemia, a treatment threshold, or incremental predictive performance of peak iSHR over absolute peak glucose. Results apply to a selected subgroup with both recent HbA1c and anesthesia-period glucose testing.
