# Supplementary material for: Early Anesthesia-Period Stress Hyperglycemia Ratio and In-Hospital Mortality After Noncardiac Surgery in Patients with HbA1c and Glucose Measurements
Source: J Clin Med. 2026 Oct 15;15(20):8002. doi: 10.3390/jcm15208002 (PMC13706281; doi:10.3390/jcm15208002)
Supplement: Supplementary file 1 [file jcm-15-08002-s001.zip › Revision Analysis Addendum.pdf]

Revision Analysis Addendum

Early Anesthesia-Period Stress Hyperglycemia Ratio and In-Hospital Mortality after Noncardiac Surgery

Decision recorded: 2026-09-20T22:00:12.837726+08:00

This addendum supplements, rather than replaces, the unchanged original Statistical Analysis Plan. It documents a reviewer-triggered revision after the original early and peak findings were already known. It is not a preregistration and does not claim blindness to prior outcomes. The original freeze record remains unchanged; subsequent implementation corrections are listed separately.

Primary exposure and population

Name: first-60-minute iSHR

Rule: first finite median-collapsed glucose in [anstart,min(anstart+60 minutes,anend)] divided by eAG from the most recent HbA1c in the preceding 90 days

N: 10406

Events: 101

Reason: The first value in a fixed early window reduces dependence on whole-anesthesia maximum-search opportunity. It does not eliminate selective measurement or establish a pretreatment exposure.

Known loss: 968 admissions, 3 deaths; their first intra-anesthesia glucose was after 60 minutes. All 113 shorter-than-60-minute operations were early evaluable.

Retained peak exposure

Whole-anesthesia peak iSHR; retain all valid original analyses. Compare early and peak on the same early-evaluable rows, adjustment set and per-0.10 scale.

Mortality endpoint

All-cause death after anesthesia end and before discharge from the same admission; time zero remains anesthesia end. Do not merge incompletely ascertained allcause_death_time records into complete 30-day mortality.

Core adjustment

age per decade

male sex

ASA >=III and missing indicators (I-II reference)

emergency

preoperative diabetes

HbA1c centered

log2 anesthesia duration

orthopedic/neurosurgery/other specialty indicators (general surgery reference)

time from anesthesia start to first glucose, per 10 minutes

Scientific interpretation

The original clinical covariates and first-glucose timing were retained without outcome-derived variable selection. Outcome-blind procedure-code mapping was assessed separately. The completed assessment supports partial AHRQ candidate classification but not a reliable surgical-severity covariate; no complexity adjustment was performed.

Procedure-severity mapping

The public release provides one five-character PCS code per operation. CMS FY2016/2018/2020, SORT/AXA, and AHRQ classifications were examined. Under the strict FY2020 rule, 5,345 of 10,406 early admissions had uniform, fully covered AHRQ candidate categories; 5,061 remained unknown. AHRQ categories describe procedural purpose and expected operating-room use rather than SORT severity. The source coding version and multiple-procedure selection rule were not documented in the available release. The assessment is concluded for this revision with a documented data limitation; no complexity-adjusted or procedure-class sensitivity model was fitted. The original frozen specification remains unchanged.

Model forms and variance

Primary: logistic three-knot RCS at early-specific 10th,50th,90th percentiles; reference 1.00, contrasts 1.20 and 1.50; 14 nonintercept parameters

Linear summary: separate linear iSHR term per 0.10; 13 nonintercept parameters

Variance: patient-clustered HC1 sandwich; same rows/knots for matched core vs expanded

Important expanded models

Add log2 whole-anesthesia glucose count, log2 baseline creatinine with original median replacement and missingness indicator, pre-anesthesia malignancy, any anesthesia-period blood-product record, and TWA65 deficit. Core and expanded RCS/linear models share the MAP-evaluable subset. TWA55 and alternative low-MAP processing are linear checks. On identical rows retain all other terms when comparing reading proportion vs TWA65.

Information timing

Duration, total count, hypotension and blood products summarize the whole anesthesia period. Associations and performance are conditional on information available by anesthesia end, not early real-time prediction or causal total/direct effects.

Missing data

No imputation of exposure components. ASA missing category as before. Creatinine median plus indicator retained as an explicit working model, with matched current full-model complete-case checks; remove the constant missingness indicator. Complete-case agreement is not proof of missing-at-random. Multiple imputation is not automatically required; reconsider only if observed instability and defensible auxiliary information justify a separately frozen model.

Sparse-event checks

Pair MLE and brglm2 AS_mean (Firth logistic mean-bias reduction) for early core and expanded RCS/linear on original fixed first-eligible-admission rows intersecting each analysis eligibility. No replacement with later admissions. Model-based Wald intervals; not profile likelihood or cluster intervals.

Fixed-horizon outcomes

Use that fixed first-patient early intersection; descriptive 7/30-day cumulative incidence with live discharge competing. Thirty-day cause-specific Cox and Fine-Gray with the early core linear terms; 7-day events are not used for a multivariable model.

Performance and validation

Same early cohort, outcome and common background (core minus separate HbA1c plus log2 total glucose count): three linear expressions absolute first glucose; first-60-minute iSHR; first glucose plus eAG. Seed 260920, 1000 shared patient-cluster bootstrap draws; fit on each bootstrap, evaluate training and original data, subtract mean optimism. Record failures and paired valid replicates. Apparent difference CI is not corrected difference CI. No LRT between nonnested expressions. Main and expanded RCS receive separate 500-resample internal calibration checks, not substituted by comparison-model calibration.

Measurement reliability

Hemoglobin: On rows with closest prior Hb in the 30 days before HbA1c, compare same-row early core with and without Hb<13 g/dL. This uses the original threshold, not a new sex-specific WHO anemia definition; report the prior surgery-anchored analysis separately.

Rbc: Exclude known RBC-positive records in 90 days before HbA1c in an exploratory recorded-exposure restriction. This reflects the approximate glycation window and is not a complete transfusion history; unknown remains unknown and is not labelled unexposed. Do not run redundant 7/30/90 association models.

Renal: Use source-confirmed N18 diagnosis/CRRT flags as documented broad renal-disease restriction, not advanced CKD staging. For single derived eGFR<30 use the available early rows and a matched core comparator; do not call it confirmed chronic disease.

Glucose source: Only source-identified classes may be modeled. If specimen/device/source is absent, do not infer POC from table location.

Hypoglycemia: Count released values below70 and54 separately from identifiability of true clinical hypoglycemia under representative-value anonymization; zero released threshold counts are not clinical absence.

Influence diagnostics

For four current core/expanded linear/RCS fits, inspect convergence/rank, fitted probabilities, leverage, Cook distance and term-specific DFBETAs. Select the top3 distinct patients by exposure-term DFBETA plus the top1 by any-term DFBETA (union) without looking at significance changes. Verify units/times/eAG/duplicates against frozen records; limited leave-whole-patient refits are diagnostic only, never automatic deletion. Report effect/CI and maximum log-OR curve change.

Multiplicity and interpretation

Overall revised RCS mortality association is the focal test. The original SAP remains unchanged; revision and all secondary/exploratory findings use nominal P values without multiplicity-adjusted confirmatory claims. No result-driven subgroups.

Implementation corrections and limits

A technical output-binding failure in the first new core run was corrected and the deterministic new-specification run was repeated; historical fits were not rerun. In the reliability output layer, an erroneous second 0.10 scaling was removed because the model term already represented a 0.10 increase. This corrected exported estimates, not model fits or populations.

The HbA1c-anchored hemoglobin audit initially reconstructed sample time from a time difference. Sub-minute floating error excluded some same-time Hb records. The final reliability analysis uses the exact raw HbA1c timestamp with the unchanged inclusive 30-day rule. The old feature and estimates remain traceable; the corrected feature and affected sensitivity outputs have distinct names and hashes. The core mortality, performance and competing-risk populations are unaffected.

Code-level and source review did not support a reliable procedure-severity variable. The available partial classification and missing source details are documented in the Supplementary Methods and the response to Reviewer 2, Comment 10. No severity-adjusted regression was performed in this revision.

Methodological sources

Nathan et al. Diabetes Care 2008;31:1473-1478. doi:10.2337/dc08-0545

Harton et al. JAMIA 2022;29:1191-1199. doi:10.1093/jamia/ocac050

Protopapa et al. BJS 2014;101:1774-1783. doi:10.1002/bjs.9638

Steyerberg et al. J Clin Epidemiol 2001;54:774-781. doi:10.1016/S0895-4356(01)00341-9

Linked revision addendum — 21 September 2026

Additional linked specifications were frozen on 21 September 2026 at 21:23:16 +08:00, after the original and revised mortality results were known and before the new secondary-outcome, monitoring-interaction and conditional-change effects were calculated. Existing mortality models and bootstrap validations were reused without alteration. The original SAP was preserved.

The early cohort was intersected with each verified outcome-specific risk set. ICU status at anesthesia end determined ICU eligibility. AKI eligibility separated baseline availability, baseline exclusions and postoperative creatinine observation; missing postoperative values were not treated as no AKI. Early outcome models retained the association core including HbA1c and first-sample offset. ICU used linear iSHR with a separate glucose-count-adjusted model; AKI and MI/stroke used the fixed early spline knots and separate linear summaries. All intervals used patient-clustered HC1 covariance. Convergence, rank, separation and tail support are summarized in Table S12; outcome-specific observation selection is in Table S11. No previous peak-cohort observation weights were applied to early AKI.

Figure 3, conditional glucose/eAG coefficients and the difference-restriction test use the same saved early comparison-model coefficients. The patient-clustered covariance was recovered from the unchanged design and score contributions; predictions matched the archived values to numerical precision. No bootstrap was repeated. The joint model includes first glucose and eAG, each per 20 mg/dL, and the common clinical background excluding HbA1c but including total glucose count. Pointwise standardized-risk intervals were computed by the delta method on the logit of the mean predicted risk. The displayed 5th–95th percentile range was set from the observed first-glucose distribution. No glucose-by-eAG interaction was fitted (Table S13).

The early monitoring analysis used whole-anesthesia categories of 1, 2 and ≥3 measurements, not the early-window measurement count. One linear model included the category main effects, early iSHR and two product terms with the current clinical core. Category-specific slopes were contrasts from that common model; the joint interaction test had two degrees of freedom. Continuous log2 count was not added to this categorical interaction model (Table S14).

Conditional glucose-change analyses included only admissions with a measurement strictly later than the first early glucose. The highest later value minus the first value could be negative. Models with core covariates plus the first glucose and with the same terms plus subsequent rise used identical admissions. The rise was scaled per 20 mg/dL. A separate sensitivity model additionally adjusted for log2 whole-anesthesia count. Initial glucose, later peak and their difference were not all entered

together. The model without initial-glucose adjustment is described in the Table S15 note; the conditional models are in Table S15.

T06: CMS/AHRQ-compatible mapping was assessed without outcome information. The strict candidate rule covered 5,345/10,406 early admissions; 5,061 remained unknown. AHRQ classes were distinguished from severity grades. The available release did not document index-procedure provenance, source version, or multiple-procedure selection, so no complexity-adjusted regression was performed. This source assessment is concluded with a documented data limitation. Unchanged mortality, complete-case, Firth, fixed-time and bootstrap results were reused after row, feature and source checks.

Model specifications were frozen at 2026-09-21T21:23:16.218550+08:00. Specification SHA-256: 70f2b5147c9df601108361ad9fcc49c6d4ed6904870217a702a2fd0b799a4870. Original and prior revision results were already known. This dated addendum documents a revision-stage analysis, not prospective registration. Source and model identity are documented in this addendum, the accompanying manuscript, Supplementary Materials, and the unchanged original Statistical Analysis Plan.
