# Supplementary material for: Early Anesthesia-Period Stress Hyperglycemia Ratio and In-Hospital Mortality After Noncardiac Surgery in Patients with HbA1c and Glucose Measurements
Source: J Clin Med. 2026 Oct 15;15(20):8002. doi: 10.3390/jcm15208002 (PMC13706281; doi:10.3390/jcm15208002)
Supplement: Supplementary file 1 [file jcm-15-08002-s001.zip › STROBE Checklist.pdf]

STROBE Checklist

Cohort study

Item	Recommendation	Location in manuscript
1	Indicate study design in title or abstract	Abstract (retrospective cohort); title
2–3	Background and objectives	Introduction, final two paragraphs
4	Present key elements of study design	Methods 2.1; revision addendum chronology
5	Setting, location, and dates	Methods 2.1 (INSPIRE v1.4.2; 2011–2020; Seoul)
6	Eligibility and selection	Methods 2.2; Figure 1; Tables S1, S2
7	Variables and definitions	Methods 2.3–2.5; Supplementary Methods; Tables S4–S10
8	Data sources and measurement	Methods 2.1–2.5; Supplementary Methods; Table S8
9	Address potential bias	Methods 2.5–2.6; Table 1; Supplementary Methods; Tables S2, S4–S9; Discussion
10	Explain study size	Methods 2.2: all eligible available records; Figure 1
11	Explain handling of quantitative variables	Methods 2.3, 2.5–2.6; Table 2; exact spline knots in Supplementary Methods
12	Describe statistical methods	Methods 2.6; Supplementary Methods; Tables S4–S10; original SAP and dated revision addendum
13	Report participant flow	Results 3.1; Figure 1; Tables S1, S2
14	Report descriptive data	Main Table 1; Tables S2, S3, and S5
15	Report outcome data	Results 3.1–3.5; Tables 2 and 4; Tables S6, S11, and S12
16	Report main and adjusted estimates	Results 3.2–3.3; Tables 2–3; Figures 2–3; Tables S4–S10
17	Report other analyses	Results 3.4–3.5; Table 5; Tables S1–S18; Figures S1–S6
18	Summarize key results	Discussion, opening paragraph
19	Discuss limitations	Discussion limitations; Methods 2.5–2.6; Supplementary Methods; Tables S8 and S9
20	Provide cautious interpretation	Discussion and Conclusions: attenuation, uncertainty and no causal/superiority inference

Item	Recommendation	Location in manuscript
21	Discuss generalizability	Abstract / Methods 2.2 / Discussion: clinically tested selected subgroup
22	Give source and role of funding	Funding statement (unchanged)

Locations refer to the current manuscript and article Supplementary Materials.

Early secondary risk sets and models are in Tables S11–S12 and Figure S5; joint glycemia, monitoring, and conditional-change analyses are in Tables S13–S15. Essential peak-iSHR sensitivity and performance results are in Tables S16–S18.
