# Supplementary material for: Early Anesthesia-Period Stress Hyperglycemia Ratio and In-Hospital Mortality After Noncardiac Surgery in Patients with HbA1c and Glucose Measurements
Source: J Clin Med. 2026 Oct 15;15(20):8002. doi: 10.3390/jcm15208002 (PMC13706281; doi:10.3390/jcm15208002)
Supplement: Supplementary file 1 [file jcm-15-08002-s001.zip › Supplementary Materials.pdf]

Supplementary Materials

Early Anesthesia-Period Stress Hyperglycemia Ratio and In-Hospital Mortality after Noncardiac Surgery in Patients with HbA1c and Glucose Measurements

Tables S1–S18 and Figures S1–S6 supplement the early-iSHR analyses and essential whole-anesthesia peak-iSHR comparisons. Reference numbers follow the main manuscript.

Supplementary Methods

Cohort and measurement rules

Operations were grouped by admission and sorted by operation date, ordering time (anesthesia start, operating-room entry, then operation start as available), and operation ID; the first operation was retained. Adults in general surgery, orthopedics, ophthalmology, obstetrics and gynecology, urology, otorhinolaryngology, neurosurgery, or plastic surgery were eligible. Complete, positive-duration anesthesia and operation intervals were required. Anesthesia start had to be within −240 to +30 min of operation start, and anesthesia end within −30 to +240 min of operation end. Different admissions from the same patient were retained (Table S1). In a separate whole-anesthesia peak-cohort enrollment-weighting diagnostic, selected admissions were compared with all 117,429 source admissions (including selected admissions). The log2-duration SMD was 0.952 before and 0.355 after weighting, and the weighted effective sample size was 4,234. Balance criteria were not met, so weighted mortality estimates were not used for inference.

HbA1c was the most recent result within 90 days before anesthesia. Patient-time duplicate glucose records were collapsed by their median. Early iSHR used the first glucose in [anesthesia start, min(anesthesia start+60 min, anesthesia end)], and peak iSHR used the highest observed anesthesia-period glucose, both divided by $eAG = 28.7 \times HbA1c - 46.7$ [23]. Glucose and eAG were expressed in mg/dL. At least one anesthesia-period measurement was sufficient for peak-cohort eligibility; the ≥2-measurement restriction was a sensitivity analysis (Table S16).

In the early cohort, 2,971 admissions had only one measurement during the entire anesthesia period, and early and peak iSHR necessarily coincided. Overall, the indices were equal in 4,748 of 10,406 admissions (45.6%). One measurement in the early window does not imply one measurement throughout anesthesia: 5,331 of the 9,967 admissions with a single early

measurement had a higher later peak. Sampling distributions and paired comparisons are provided in Tables S3, S2, and S4.

Outcome ascertainment

Mortality follow-up began at anesthesia end and ended at death or discharge from the same admission. The separate allcause_death_time field did not establish complete ascertainment of deaths after discharge within 30 days of anesthesia end: among 10,782 first-eligible whole-anesthesia peak admissions, 42 death dates within 30 days of anesthesia end were all after discharge, while 8,386 records had no value in that field. The two death timestamps differed in records with both fields populated. The fields were therefore not combined to classify 30-day all-cause survival. Fixed-horizon analyses used the first-eligible-admission subset, with live discharge as a competing event. The ICU risk set excluded patients already in ICU at anesthesia end or with indeterminate status. Eligible entries were after anesthesia end, within 24 h, and no later than discharge. The surgery-anchored release window limits ascertainment and does not distinguish planned from unplanned ICU admission. The early ICU risk set contained 10,216 admissions and 1,882 eligible records (Table S11). Among 117,429 source admissions, one death was recorded before or at anesthesia end and did not enter the exposure cohorts.

New myocardial infarction or stroke records were identified using I21–I22 or I60–I64 between anesthesia end and discharge or valid in-hospital death. Corresponding diagnoses from admission through anesthesia end were excluded. Diagnosis-record time was used as the event time. The composite did not include death.

For AKI, the most recent creatinine within 7 days before anesthesia was required to be >0 and <4.5 mg/dL. Admissions with pre-anesthesia renal replacement therapy or the specified preoperative creatinine-rise pattern were excluded. An increase from baseline of ≥0.3 mg/dL within 48 h or ≥1.5-fold within 7 days after anesthesia end, before discharge, identified AKI [24]. Postoperative creatinine availability was required; missing measurements were not coded as no AKI. In the whole-anesthesia peak-iSHR cohort, 7,133 admissions had baseline creatinine; sequential exclusions were 335 for an invalid or out-of-range baseline, 24 with preoperative renal replacement therapy, 73 with the specified preoperative creatinine-rise pattern, and 396 without an eligible postoperative creatinine observation, leaving 6,305 admissions with 434 AKI events. Urine-output criteria were not used because longitudinal observations sufficient for timed,

weight-normalized assessment were not established; AKI meeting urine-output criteria alone may therefore be missed. The early-iSHR AKI eligibility flow is summarized in Table S11.

Covariates and hypotension burden

ASA was coded as I–II, ≥III, or missing; specialty as general surgery, orthopedics, neurosurgery, or other. Age was scaled per 10 years, sample offset per 10 min, and anesthesia duration log2-transformed. Count used log2(count/2). Creatinine was log2(value/median), with the observed peak-cohort median used for replacement and a separate missingness indicator. Malignancy was any C00–C99 record at or before anesthesia start, without a fixed look-back or same-admission restriction. Blood products indicated any positive rbc, ffp, pc, pheresis, or cryo record during anesthesia.

MAP observations were restricted to 20–200 mmHg before within-source/time median aggregation, preferring arterial over noninvasive values. Observations ≤10 min apart were joined by linear interpolation, with explicit threshold-crossing integration; longer gaps and window edges were not extrapolated. TWA65 or TWA55 was positive deficit area divided by connected observation time. At least 80% coverage of anesthesia duration was required. A positive-finite-MAP alternative was compared on common eligible records. The low-MAP reading proportion and TWA specifications are separately labeled (Tables S4, S17).

Association models and sensitivity analyses

Early-iSHR spline knots were 0.69410913, 0.86776860, and 1.12151356; peak-iSHR knots were 0.74783828, 0.99611043, and 1.44455578. Knots remained fixed within exposure-specific subsets. The early core model included first-sample offset in addition to the peak core covariates. Early core/expanded RCS models had 14/20 nonintercept parameters; their linear versions had 13/19. Peak core RCS/linear models had 13/12. Association intervals used patient-clustered HC1 covariance; iSHR contrasts were referenced to 1.00. Full early model coefficients are in Table S10.

Creatinine-complete analyses compared core and expanded models on identical observations and omitted the constant missingness indicator. For Firth/MLE comparisons, each patient's first whole-anesthesia peak-iSHR-eligible admission was selected and then restricted to records meeting early-iSHR and MAP eligibility. Firth used mean-bias reduction (AS_mean) and model-based Wald intervals [26]. Influence analyses separately excluded each of the distinct patients with the three largest exposure-term |DFBETA| values and the largest |DFBETA| across all terms, retaining fixed knots. Primary estimates retained all eligible admissions (Tables S5, S9).

HbA1c-reliability analyses used the most recent hemoglobin at or before HbA1c sampling within 30 days, with Hb<13 g/dL as a common sensitivity threshold. An RBC-positive signal was a patient-linked vitals entry labeled 'rbc' with value >0 in the 90 days before HbA1c sampling; other blood products were excluded from this indicator. A single eGFR<30 mL/min/1.73 m² was a renal-function proxy. Missing source records did not establish absence of prior transfusion or advanced renal disease. The INSPIRE data descriptor reports that intraoperative laboratory tests were mainly point-of-care arterial blood gas analyses in patients with arterial catheters [21]. The released glucose records lack a testing-method field; proportions by test source cannot be calculated (Table S8). Clinical hypoglycemia cannot be excluded from anonymized representative values.

The monitoring interaction model used whole-anesthesia counts of 1, 2, and ≥3, their main effects, and interactions with linear early iSHR. The joint interaction test had two degrees of freedom. Conditional change analyses required a measurement strictly after the first early glucose; change was the highest later glucose minus the first value, retaining negative values. Common-case models adjusted for first glucose, with further log2 count adjustment assessed separately (Tables S14, S15).

Secondary outcomes and fixed-horizon mortality

ICU models used linear early iSHR, with and without glucose-count adjustment. AKI and myocardial infarction/stroke models used fixed early spline knots and separate linear summaries in their own risk sets. Postoperative creatinine availability was compared within the baseline-eligible early cohort. In the first-admission early subset, cumulative incidence treated live discharge as a competing event, with administrative censoring at day 30. Day-7 estimates were descriptive; day-30 cause-specific Cox and Fine–Gray models used core linear covariates, with proportional-hazards checks (Tables S6, S11, S12).

Joint glycemia models and internal performance

Joint models included glucose and eAG per 20 mg/dL, with clinical core covariates except the separate HbA1c term, plus total count. Risks at eAG 100, 120, and 150 mg/dL were standardized over other covariates; pointwise intervals used the delta method and patient-clustered covariance. The displayed first-glucose range was the 5th–95th percentiles. Testing whether the untransformed glucose and eAG coefficients summed to zero assessed a glucose-difference restriction (Table S13).

Performance comparisons used absolute glucose, iSHR, or glucose plus eAG with identical cases and common clinical covariates excluding HbA1c and including count. Each of 1,000 patient-cluster bootstrap samples was shared across the three models and retained all admissions of sampled patients. Mean optimism corrected point estimates; percentile intervals for paired apparent differences were reported separately [25]. Early association RCS models each used 500 bootstrap repetitions for internal calibration. C-statistics, Brier scores, calibration and fit measures are reported in Tables S7, S9, and S18. The absolute-glucose model was nested within the glucose-plus-eAG model, whereas the iSHR model was nonnested with either alternative.

Analysis documentation

The statistical analysis plan and a dated amendment document the analysis sequence, including designation of first-60-minute iSHR as the primary exposure. The whole-anesthesia peak-iSHR models used NumPy for estimation and pandas for data preparation. These models were independently reproduced from the frozen cohort under Python 3.12.14, NumPy 2.3.5 and pandas 3.0.1, with numerical agreement across the saved results. Survival and bias-reduced analyses used R 4.3.0 with survival 3.5-5 and brglm2 0.9.2; the linked early models used R 4.6.1.

Supplementary Tables

Table S1: Cohort assembly and key data availability (surgical-admission and patient levels)

Step or measure	Records	Proportion/denominator
Panel A Sequential peak-iSHR cohort (surgical-admission level)		
All operation records	130,960	100.00%
First operation per admission	126,673	96.73%
Strict adult noncardiac surgery	117,957	90.07%
Valid anesthesia window	117,643	89.83%
Time-consistent cohort (source cohort)	117,429	89.67%
Eligible HbA1c + anesthesia-period glucose (peak-iSHR cohort)	11,374	9.69%*

Step or measure	Records	Proportion/denominator
Panel B Parallel availability in the source cohort (N=117,429)		
Eligible HbA1c available	27,555	23.47%
Anesthesia-period glucose available	34,050	29.00%
Both measures available	11,374	9.69%
Panel C Patient-level robustness cohort (unique patients)		
Earliest exposure-eligible operation per patient	10,782	94.79%†
≥2 anesthesia-period glucose measurements	7,543	69.96%‡
≥3 anesthesia-period glucose measurements	4,045	37.52%‡
≥4 anesthesia-period glucose measurements	2,179	20.21%‡

Panel A gives the sequential surgical-admission flow, and Panel B measurement availability among the same 117,429 source admissions. Panel C uses the first eligible admission of each of 10,782 patients from the 11,374 eligible admissions. *11,374/117,429; †10,782/11,374; ‡counts divided by 10,782. Repeated-measurement counts in this panel therefore use a different denominator from the all-admission analyses.

Table S2. Early glucose availability and sampling window

Early-window evidence	Available	Unavailable / comparator
Admissions / deaths	10,406 / 101	968 / 3
Unique patients	9,888	966 (groups can share patients)
First sample offset, median (IQR), min	25 (20–40)	80 (range 65–425)
Anesthesia <60 min	113; all early-evaluable	0 exclusions
Anesthesia duration, median (IQR), min	195 (135–285)	250 (175–335)
Early count 1 / 2 / ≥3	9,967 / 419 / 20	Not early-evaluable
Fixed first-eligible-admission intersection	9,849 / 87 deaths	39 early-evaluable patients had an earlier ineligible admission

The first-60-minute and whole-anesthesia peak analyses used distinct eligibility windows; 968 admissions lacked a qualifying first-60-minute exposure. Measurement counts refer to their respective windows. Selection from all source admissions is shown in Table 1.

Table S3. Glucose sampling by mortality status and exposure quartile

Early sampling and first-sample timing

Group	Admissions	Patients	Deaths	Whole-anesthesia glucose count, median (IQR)	Early-window glucose count, median (IQR)	First early-sample offset, median (IQR), min
All early-iSHR admissions	10,406	9,888	101	2.000 (1.000–3.000)	1.000 (1.000–1.000)	25.000 (20.000–40.000)
No in-hospital death	10,305	9,800	0	2.000 (1.000–3.000)	1.000 (1.000–1.000)	25.000 (20.000–40.000)
In-hospital death	101	101	101	3.000 (2.000–5.000)	1.000 (1.000–1.000)	25.000 (20.000–40.000)
Early iSHR Q1	2,622	2,537	22	2.000 (1.000–3.000)	1.000 (1.000–1.000)	25.000 (20.000–35.000)
Early iSHR Q2	2,765	2,733	14	2.000 (1.000–3.000)	1.000 (1.000–1.000)	25.000 (15.000–35.000)
Early iSHR Q3	2,459	2,425	19	2.000 (1.000–3.000)	1.000 (1.000–1.000)	25.000 (20.000–35.000)
Early iSHR Q4	2,560	2,507	46	2.000 (1.000–3.000)	1.000 (1.000–1.000)	30.000 (20.000–40.000)

Counts are descriptive. The early window is from anesthesia start through 60 minutes (or anesthesia end if earlier); whole-anesthesia counts use the full anesthesia interval. Early iSHR quartiles are defined within the early cohort and are not outcome-derived model strata.

Panel B. Whole-anesthesia peak-iSHR cohort

Group	Admissions	Measurements	Measurements per hour	First after start, min	Last before end, min	Peak after start, min
Survived	11,270	2 (1–3)	0.63 (0.49–0.83)	30 (20–40)	65 (40–95)	90 (30–155)
Died during same admission	104	3 (2–5)	0.94 (0.69–1.17)	25 (20–40)	45 (30–65)	47.5 (23.8–161.3)

Group	Admissions	Measurements	Measurements per hour	First after start, min	Last before end, min	Peak after start, min
Peak iSHR Q1	2,850	1 (1–2)	0.59 (0.46–0.77)	25 (20–40)	75 (50–105)	35 (20–75)
Peak iSHR Q2	2,886	2 (1–3)	0.62 (0.48–0.78)	25 (20–40)	70 (40–100)	50 (25–125)
Peak iSHR Q3	2,834	2 (2–3)	0.67 (0.51–0.84)	30 (20–40)	60 (35–90)	110 (45–160)
Peak iSHR Q4	2,804	3 (2–4)	0.70 (0.53–0.90)	30 (20–45)	55 (30–85)	150 (100–225)

Values are medians (interquartile ranges). Panel A uses the early cohort and distinguishes whole-anesthesia from early-window counts; Panel B retains the peak cohort. Sampling was clinically determined. The early common monitoring-interaction model is in Table S14 and Figure S1.

Table S4. Early iSHR and same-cohort peak iSHR associations with mortality

Model	N / deaths	df*	OR per 0.10 (95% CI)	Overall P	Nonlinear P
Early iSHR: core, linear	10,406 / 101	13	1.134 (1.063–1.210)	1.30e-04	—
Same MAP subset: core, linear	10,352 / 97	13	1.118 (1.046–1.195)	0.001	—
TWA65 + count expanded, linear	10,352 / 97	19	1.089 (1.014–1.170)	0.019	—
Early iSHR: core, RCS	10,406 / 101	14	RCS; contrasts in Table 2	5.97e-05	0.304
Same MAP subset: core, RCS	10,352 / 97	14	RCS; contrasts in Table 2	5.68e-04	0.291
TWA65 + count expanded, RCS	10,352 / 97	20	RCS; contrasts in Table 2	0.047	0.689
Early iSHR: core + count	10,406 / 101	14	1.119 (1.047–1.196)	8.82e-04	—
Same MAP subset: core + count	10,352 / 97	14	1.100 (1.027–1.179)	0.007	—

Model	N / deaths	df*	OR per 0.10 (95% CI)	Overall P	Nonlinear P
Expanded with low-MAP reading proportion	10,352 / 97	19	1.085 (1.010–1.164)	0.025	—
Expanded with TWA55	10,352 / 97	19	1.094 (1.018–1.175)	0.014	—
Expanded with alternative-rule TWA65	10,352 / 97	19	1.093 (1.018–1.173)	0.014	—
Expanded with alternative-rule TWA55	10,352 / 97	19	1.097 (1.022–1.178)	0.010	—
Peak iSHR on early rows: matched core	10,406 / 101	13	1.123 (1.059–1.190)	9.49e-05	—
Peak iSHR on early rows: matched core + count	10,406 / 101	14	1.083 (1.018–1.152)	0.011	—

*df excludes the intercept. All intervals use patient-clustered HC1 sandwich variance. RCS uses the frozen early knots and two exposure terms; linear models use per 0.10. Matched adjustment comparisons use identical MAP-eligible observations within each analysis. Table S17 reports whole-anesthesia peak-iSHR models.

Table S5. Early and whole-anesthesia peak iSHR in matched Firth and creatinine-complete analyses

Specification / estimator	N / deaths	df*	Linear OR or RCS 1.50 vs 1.00 (95% CI)	Overall P	Nonlinear P
Creatinine complete: core, linear; MLE	6,505 / 88	13	1.105 (1.029–1.186)	0.006	—
Creatinine complete: expanded, linear; MLE	6,505 / 88	18	1.071 (0.994–1.154)	0.070	—
Creatinine complete: core, RCS; MLE	6,505 / 88	14	1.875 (1.256–2.800)	0.005	0.339
Creatinine complete: expanded, RCS; MLE	6,505 / 88	19	1.452 (0.925–2.278)	0.173	0.843
Peak iSHR: creatinine-complete core, linear; MLE	7,133 / 94	12	1.118 (1.053–1.187)	2.44e-04	—
Peak iSHR: creatinine-complete low-MAP-proportion-expanded (no count), linear; MLE	7,133 / 94	16	1.055 (0.989–1.125)	0.102	—
Peak iSHR: MAP/creatinine-complete core on TWA-eligible records; MLE	7,093 / 90	12	1.105 (1.041–1.174)	0.001	—
Peak iSHR: MAP/creatinine-complete TWA65/count-expanded; MLE	7,093 / 90	17	1.026 (0.960–1.097)	0.447	—

Specification / estimator	N / deaths	df*	Linear OR or RCS 1.50 vs 1.00 (95% CI)	Overall P	Nonlinear P
Early iSHR: core, linear — first-eligible-admission subset; MLE	9,849 / 87	13	1.165 (1.091–1.244)	5.68e-06	—
Early iSHR: core, linear — first-eligible-admission subset; Firth_AS_mean	9,849 / 87	13	1.164 (1.091–1.241)	3.91e-06	—
Early iSHR: core, RCS — first-eligible-admission subset; MLE	9,849 / 87	14	2.283 (1.532–3.402)	2.06e-05	0.599
Early iSHR: core, RCS — first-eligible-admission subset; Firth_AS_mean	9,849 / 87	14	2.281 (1.545–3.367)	1.47e-05	0.535
TWA65 + count expanded, linear — first-eligible-admission subset; MLE	9,800 / 84	19	1.117 (1.041–1.199)	0.002	—
TWA65 + count expanded, linear — first-eligible-admission subset; Firth_AS_mean	9,800 / 84	19	1.116 (1.042–1.194)	0.002	—
TWA65 + count expanded, RCS — first-eligible-admission subset; MLE	9,800 / 84	20	1.740 (1.120–2.701)	0.009	0.995
TWA65 + count expanded, RCS — first-eligible-admission subset; Firth_AS_mean	9,800 / 84	20	1.742 (1.139–2.664)	0.007	0.937

Firth_AS_mean is mean-bias-reduced logistic estimation with model-based Wald intervals, paired with MLE on the same first-eligible-admission rows and fixed knots. Creatinine-complete analyses omit the constant creatinine-missingness indicator and use patient-clustered intervals. For whole-anesthesia peak iSHR, the creatinine-complete core and low-MAP-proportion-expanded models used 7,133 admissions and 94 deaths; the separate TWA65/count MAP-eligible models used 7,093 admissions and 90 deaths. In the peak first-eligible-admission subset (10,782 admissions; 90 deaths), core linear MLE and Firth ORs per 0.10 were 1.144 (1.079–1.212) and 1.143 (1.080–1.209); low-MAP-proportion-expanded linear ORs were 1.070 (1.005–1.141) and 1.070 (1.006–1.137).

Table S5 (continued). Selection associated with creatinine availability

Variable	All MAP-evaluable	Creatinine complete	Creatinine missing
Admissions / deaths	10,352 / 97	6,505 / 88	3,847 / 9
Mortality, %	0.94	1.35	0.23
Diabetes, n (%)	1,722 (16.6)	1,171 (18.0)	551 (14.3)
ASA ≥III, n (%)	1,637 (15.8)	1,319 (20.3)	318 (8.3)
Emergency, n (%)	758 (7.3)	683 (10.5)	75 (1.9)

Variable	All MAP-evaluable	Creatinine complete	Creatinine missing
Malignancy record, n (%)	4,388 (42.4)	2,487 (38.2)	1,901 (49.4)
Any blood-product record, n (%)	423 (4.1)	330 (5.1)	93 (2.4)

The complete and missing groups partition the same 10,352 MAP-evaluable admissions. Patients with multiple admissions can contribute to both availability groups. Complete cases retain 88 of 97 deaths in 6,505 admissions. The complete-case analysis is presented as a sensitivity analysis, with selection related to creatinine availability described by these groups.

Table S6. Early iSHR and fixed-horizon in-hospital mortality

Measure	N / events	Estimate (95% CI) or result
7-day in-hospital death CIF	9,849 / 22	0.223%
30-day in-hospital death CIF	9,849 / 56	0.569%
30-day discharge / remaining in hospital	9,396 / 397	Live discharge is a competing event
30-day cause-specific HR per 0.10	9,849 / 56	1.159 (1.074–1.250)
30-day subdistribution HR per 0.10	9,849 / 56	1.196 (1.104–1.296)
Cox proportionality check	Exposure / global P	0.168 / 0.157
Fine–Gray proportionality check	Exposure / global P	0.343 / 0.439

Among each patient's first whole-anesthesia peak-iSHR-eligible admission, the fixed-horizon analysis included those also meeting early-iSHR eligibility. Time zero is anesthesia end. Cox uses ordinary model-based intervals; Fine–Gray uses robust intervals for weighted expanded records. Both have 13 nonintercept covariates including the exposure and no TWA expansion. Individual nuisance-term proportionality checks had nominal $P<0.05$ for age, emergency and duration in Cox, and ASA≥III in Fine–Gray; global and exposure tests did not. These sensitivity models concern in-hospital events through 30 days. For descriptive context, the full early cohort comprised 10,406 admissions and 101 deaths: median follow-up to death or discharge was 6.5 days (IQR 4.4–10.3), median time to death 20.4 days (7.2–56.3), and 38 deaths occurred after day 30. Median time to live discharge was 6.5 days (4.4–10.3) among 10,305 survivors. These full-cohort summaries are separate from the fixed-first-patient models above. In the whole-anesthesia peak-iSHR first-eligible-admission subset, the 30-day in-hospital cause-specific Cox analysis included 10,782 admissions and 58 deaths (HR 1.122, 95% CI 1.047–1.201 per 0.10).

Table S7. Full early-expression internal performance comparison

Expression / metric	Apparent	Apparent 95% CI	Corrected point	Valid bootstrap n
Absolute first glucose / C_statistic	0.876784	0.846707–0.914202	0.863996	1,000
Absolute first glucose / Brier	0.009093	0.007401–0.010752	0.009205	1,000

Expression / metric	Apparent	Apparent 95% CI	Corrected point	Valid bootstrap n
Absolute first glucose / Calibration_intercept	0.000000	Not reported for fitting properties	-0.005106	1,000
Absolute first glucose / Calibration_slope	1.000000	Not reported for fitting properties	0.943829	1,000
First-60-minute iSHR / C_statistic	0.879312	0.851481–0.916511	0.866407	1,000
First-60-minute iSHR / Brier	0.009068	0.007373–0.010696	0.009191	1,000
First-60-minute iSHR / Calibration_intercept	0.000000	Not reported for fitting properties	-0.004736	1,000
First-60-minute iSHR / Calibration_slope	1.000000	Not reported for fitting properties	0.945215	1,000
First glucose + eAG / C_statistic	0.880238	0.854072–0.917666	0.866415	1,000
First glucose + eAG / Brier	0.009092	0.007368–0.010736	0.009223	1,000
First glucose + eAG / Calibration_intercept	0.000000	Not reported for fitting properties	-0.005270	1,000
First glucose + eAG / Calibration_slope	1.000000	Not reported for fitting properties	0.941020	1,000

Training-minus-apparent-sample optimism was estimated from 1,000 shared patient-cluster bootstrap draws. Intercepts and slopes near 0 and 1 in training are fitting properties; no degenerate training CI is reported. Corrected metrics are points, not external validation. Main Table 3 provides fit criteria. The separate association-RCS calibration results appear in Table S9.

Table S7 (continued). Paired differences from absolute early glucose

Expression / metric	Apparent difference	95% CI for apparent difference	Paired valid n
early_iSHR minus early_glucose / C_statistic	0.002528	-0.001268–0.006885	1,000
early_iSHR minus early_glucose / Brier	-0.000025	-0.000198–0.000043	1,000

Expression / metric	Apparent difference	95% CI for apparent difference	Paired valid n
early_glucose_plus_eAG minus early_glucose / C_statistic	0.003454	-0.000133–0.009964	1,000
early_glucose_plus_eAG minus early_glucose / Brier	-0.000001	-0.000122–0.000041	1,000

These intervals use paired refitted apparent differences. They are not uncertainty intervals for optimism-corrected differences, and inclusion of zero does not establish equivalence.

Table S8. HbA1c reliability and available-data sensitivity analyses

Recorded feature / matched analysis	N / deaths	df*	OR per 0.10 (95% CI)	P value
Panel A. First-60-minute iSHR				
HbA1c-prior-30-day Hb available; early-iSHR core	8,136 / 88	13	1.134 (1.054–1.220)	7.15e-04
Same Hb30 rows; add Hb<13 indicator	8,136 / 88	14	1.132 (1.054–1.216)	6.83e-04
Baseline creatinine/eGFR available; early-iSHR core	6,537 / 92	13	1.122 (1.048–1.202)	9.14e-04
Same eGFR rows; add single-baseline eGFR<30 proxy	6,537 / 92	14	1.128 (1.055–1.207)	4.45e-04
Exclude recorded RBC-positive signal in prior 90 days; early-iSHR core	10,400 / 101	13	1.134 (1.063–1.210)	1.34e-04
Exclude recorded pre-HbA1c N18 or positive CRRT; early-iSHR core	9,751 / 75	13	1.091 (0.997–1.193)	0.057
Panel B. Whole-anesthesia peak iSHR				
Hb within 30 days before anesthesia; peak-iSHR core	10,795 / 101	12	1.130 (1.067–1.196)	2.90e-05
Same pre-anesthesia Hb rows; add Hb<13 g/dL	10,795 / 101	13	1.123 (1.063–1.187)	3.61e-05
Baseline creatinine/eGFR available; peak-iSHR core	7,133 / 94	12	1.118 (1.053–1.187)	2.44e-04
Same eGFR rows; add single-baseline eGFR<30 proxy	7,133 / 94	13	1.122 (1.059–1.189)	9.72e-05
Exclude admissions linked to patient RBC-positive signal within 90 d before HbA1c; peak-iSHR core	11,367 / 104	12	1.128 (1.065–1.194)	3.49e-05

Panel A uses first-60-minute iSHR and core covariates including first-sample offset; its hemoglobin is the nearest value at or before HbA1c sampling within 30 days. Panel B uses whole-anesthesia peak iSHR and peak core

covariates; its hemoglobin is the nearest value within 30 days before anesthesia. Both panels use patient-clustered HC1 intervals. Hb<13 g/dL is a common sensitivity threshold, and a single eGFR<30 mL/min/1.73 m² is a renal-function proxy. For both exposures, admissions were excluded when the patient had an RBC-positive record in the 90 days before the HbA1c sample. The available source does not capture complete transfusion histories.

Table S8 (continued). Field-level evidence and residual uncertainty

Source question	Whole-anesthesia peak-iSHR cohort evidence	Clinical interpretation
Hb in 30 d before HbA1c	8,895 / 91 deaths; Hb<13: 4,454 / 69	Nearest prior Hb at the exact source HbA1c time, including same-time records
RBC in 90 d before HbA1c	7 known-positive admissions; 0 deaths	No complete ward/hospital transfusion history; other admissions are clinically unknown
Generic N18 / single eGFR<30	692 / 25; 570 / 22	Broad diagnosis and single renal proxy; advanced stage and dialysis dependence unresolved
Glucose testing method	INSPIRE describes predominantly point-of-care arterial blood gas testing in patients with arterial catheters [21]; individual glucose records lack a testing-method field	Proportions by testing method cannot be calculated
Released glucose thresholds	<70 mg/dL: 0 admissions / 0 deaths; <54 mg/dL: 0 admissions / 0 deaths	True clinical hypoglycemia is not established as absent

The whole-anesthesia peak-iSHR cohort denominator is 11,374 unless stated otherwise. A negative record search is not proof that the clinical condition was absent. Panels A and B use the model-specific N and death counts reported in each row. Exact-time duplicate glucose rows were median-collapsed; rederived measurement counts matched all eligible admissions.

Table S9. Early-iSHR model diagnostics influence analyses and calibration

Model	Rank / parameters	Max leverage / Cook	Max exposure |DFBETA|	Limited patient refits
Early iSHR: core, linear	14 / 14	0.100 / 0.026	1.381	3; OR 1.121–1.125
TWA65 + count expanded, linear	20 / 20	0.114 / 0.028	1.478	3; OR 1.071–1.079

Model	Rank / parameters	Max leverage / Cook	Max exposure |DFBETA|	Limited patient refits
Early iSHR: core, RCS	15 / 15	0.140 / 0.025	0.952	4; OR 1.958–2.095
TWA65 + count expanded, RCS	21 / 21	0.121 / 0.036	1.333	3; OR 1.415–1.603

All four fits converged and had finite coefficients and no numerical boundary flag; this is not proof of absence of every form of separation. Exposure terms were examined separately from nuisance terms. Top 3 exposure-influence patients plus top 1 overall-influence patient were selected before diagnostic refits; overlaps explain 3 versus 4 refits. Linear ORs are per 0.10 and RCS ORs compare 1.50 with 1.00. No flagged patient was removed from the main analysis. Initial DFBETAs were observation-level diagnostics; actual sensitivity refits omitted whole patient clusters.

Table S9 (continued). Estimates after exclusion of selected influential patients

Model / diagnostic omission	Removed admissions / deaths	Refit OR (95% CI)	Maximum |log-OR curve change|
Early iSHR: core, linear / check 1	2 / 1	1.121 (1.047–1.201)	0.011
Early iSHR: core, linear / check 2	1 / 1	1.123 (1.052–1.200)	0.009
Early iSHR: core, linear / check 3	1 / 1	1.125 (1.052–1.202)	0.008
TWA65 + count expanded, linear / check 1	2 / 1	1.071 (0.997–1.151)	0.016
TWA65 + count expanded, linear / check 2	1 / 1	1.077 (1.000–1.161)	0.011
TWA65 + count expanded, linear / check 3	1 / 1	1.079 (1.002–1.163)	0.009
Early iSHR: core, RCS / check 1	2 / 1	1.958 (1.325–2.895)	0.037
Early iSHR: core, RCS / check 2	3 / 1	2.095 (1.470–2.985)	0.101
Early iSHR: core, RCS / check 3	1 / 1	1.991 (1.380–2.873)	0.026
Early iSHR: core, RCS / check 4	1 / 1	2.040 (1.425–2.921)	0.013
TWA65 + count expanded, RCS / check 1	2 / 1	1.415 (0.908–2.207)	0.072
TWA65 + count expanded, RCS / check 2	3 / 1	1.603 (1.036–2.481)	0.105
TWA65 + count expanded, RCS / check 3	1 / 1	1.510 (0.946–2.408)	0.033

Diagnostic labels are model-specific and refer to cases selected by the |DFBETA| rule in the Supplementary Methods. Curve changes are evaluated over the common 5th–95th percentile early-exposure range; the 1.50 contrast may lie outside that display range.

Table S9 (continued). Source-consistency checks for flagged observations

Model	Selected patients / admissions	Selected deaths	Unit/formula/time/join errors
Early iSHR: core, linear	3 / 4	3	0
Early iSHR: core, RCS	4 / 7	4	0
TWA65 + count expanded, linear	3 / 4	3	0
TWA65 + count expanded, RCS	3 / 6	3	0

Units, eAG calculation, early glucose/eAG arithmetic, anesthesia-window timing, duplicate operation rows and joins were checked against frozen cohort/input records. Across the eight distinct selected operations, 1,338 raw glucose rows collapsed to 1,332 patient-time records; all eight first-window times and values matched the raw source. This confirms source consistency, not assay accuracy or absence of clinical confounding. Flagged valid observations were retained in the primary analysis; limited cluster omissions were diagnostic only.

Table S9 (continued). Association-model internal calibration

Model / metric	Apparent	Corrected point	Valid resamples
Early iSHR: core, RCS / C_statistic	0.866662	0.850821	500
Early iSHR: core, RCS / Brier	0.009129	0.009241	500
Early iSHR: core, RCS / Calibration_intercept	-0.000000	-0.005784	500
Early iSHR: core, RCS / Calibration_slope	1.000000	0.937483	500
TWA65 + count expanded, RCS / C_statistic	0.896768	0.877051	500
TWA65 + count expanded, RCS / Brier	0.008504	0.008838	500
TWA65 + count expanded, RCS / Calibration_intercept	-0.000000	0.002437	500
TWA65 + count expanded, RCS / Calibration_slope	1.000000	0.919653	500

These are separate 500-resample checks for the main early RCS and its important TWA-expanded RCS, not the three linear performance models. Apparent intercept and slope are fitting properties; corrected values are internal-validation points, not externally validated estimates.

Table S10. Full coefficients of the early-iSHR core and expanded spline models

Model / term	Coefficient (SE)	Conditional OR (95% CI)	P value
Early iSHR: core, RCS / (Intercept)	-5.7897 (0.3755)	0.003 (0.001–0.006)	1.25e-53
Early iSHR: core, RCS / early_rcs_linear	-0.0145 (0.1426)	Basis coefficient; use exposure contrasts	0.919

Model / term	Coefficient (SE)	Conditional OR (95% CI)	P value
Early iSHR: core, RCS / early_rcs_nonlinear	1.3542 (1.3187)	Basis coefficient; use exposure contrasts	0.304
Early iSHR: core, RCS / age_decade	0.5143 (0.1203)	1.672 (1.321–2.117)	1.91e-05
Early iSHR: core, RCS / male	0.1526 (0.2187)	1.165 (0.759–1.788)	0.485
Early iSHR: core, RCS / asa_high	1.6823 (0.2586)	5.378 (3.239–8.927)	7.75e-11
Early iSHR: core, RCS / asa_missing	0.8453 (0.5652)	2.329 (0.769–7.050)	0.135
Early iSHR: core, RCS / emergency	1.3392 (0.2626)	3.816 (2.281–6.385)	3.39e-07
Early iSHR: core, RCS / diabetes	0.3417 (0.2574)	1.407 (0.850–2.331)	0.184
Early iSHR: core, RCS / hba1c_centered	-0.0546 (0.1200)	0.947 (0.748–1.198)	0.649
Early iSHR: core, RCS / log2_duration	-0.0641 (0.1708)	0.938 (0.671–1.311)	0.708
Early iSHR: core, RCS / dept_OS	-0.7694 (0.3292)	0.463 (0.243–0.883)	0.019
Early iSHR: core, RCS / dept_NS	-0.2564 (0.3063)	0.774 (0.425–1.410)	0.402
Early iSHR: core, RCS / dept_Other	-0.3285 (0.2806)	0.720 (0.415–1.248)	0.242
Early iSHR: core, RCS / first60_offset_10min	0.0294 (0.0785)	1.030 (0.883–1.201)	0.708
TWA65 + count expanded, RCS / (Intercept)	-5.7871 (0.4368)	0.003 (0.001–0.007)	4.49e-40
TWA65 + count expanded, RCS / early_rcs_linear	0.0258 (0.1533)	Basis coefficient; use exposure contrasts	0.867
TWA65 + count expanded, RCS / early_rcs_nonlinear	0.5832 (1.4595)	Basis coefficient; use exposure contrasts	0.689
TWA65 + count expanded, RCS / age_decade	0.4795 (0.1347)	1.615 (1.240–2.103)	3.71e-04
TWA65 + count expanded, RCS / male	-0.0045 (0.2354)	0.996 (0.628–1.579)	0.985
TWA65 + count expanded, RCS / asa_high	1.3368 (0.2760)	3.807 (2.216–6.539)	1.28e-06
TWA65 + count expanded, RCS / asa_missing	0.5823 (0.5938)	1.790 (0.559–5.732)	0.327
TWA65 + count expanded, RCS / emergency	0.9115 (0.3015)	2.488 (1.378–4.493)	0.003
TWA65 + count expanded, RCS / diabetes	0.2788 (0.2783)	1.321 (0.766–2.280)	0.316

Model / term	Coefficient (SE)	Conditional OR (95% CI)	P value
TWA65 + count expanded, RCS / hba1c_centered	-0.0618 (0.1203)	0.940 (0.743–1.190)	0.607
TWA65 + count expanded, RCS / log2_duration	-0.6548 (0.2705)	0.520 (0.306–0.883)	0.015
TWA65 + count expanded, RCS / dept_OS	-0.6265 (0.3779)	0.534 (0.255–1.121)	0.097
TWA65 + count expanded, RCS / dept_NS	0.0438 (0.3623)	1.045 (0.514–2.125)	0.904
TWA65 + count expanded, RCS / dept_Other	-0.1091 (0.3687)	0.897 (0.435–1.847)	0.767
TWA65 + count expanded, RCS / first60_offset_10min	-0.0498 (0.0887)	0.951 (0.800–1.132)	0.574
TWA65 + count expanded, RCS / log2_glucose_count	0.6248 (0.2418)	1.868 (1.163–3.000)	0.010
TWA65 + count expanded, RCS / log2_creatinine_centered	0.0450 (0.1489)	1.046 (0.781–1.400)	0.763
TWA65 + count expanded, RCS / creatinine_missing	-0.8894 (0.4386)	0.411 (0.174–0.971)	0.043
TWA65 + count expanded, RCS / malignancy	0.1005 (0.2683)	1.106 (0.654–1.871)	0.708
TWA65 + count expanded, RCS / any_transfusion	0.8075 (0.3633)	2.242 (1.100–4.570)	0.026
TWA65 + count expanded, RCS / twa65_deficit_mmHg_main	0.2784 (0.0561)	1.321 (1.183–1.474)	6.98e-07

RCS basis-term exponentials do not represent a constant per 0.10 exposure effect; clinical contrasts are in Table 2. Other ORs are conditional regression coefficients and are not individual causal effects. HbA1c and diabetes terms are conditional on the other model variables. All SEs in this table are patient-clustered HC1.

Table S11. Early secondary-outcome populations and observation selection

A. Outcome-specific eligibility

Stage	Admissions	Patients	Events / note
Early eligible cohort	10,406	9,888	101 deaths
ICU: exclude already in ICU	189	189	
ICU: exclude unknown prior status	1	1	
ICU: final risk set	10,216	9,710	1,882 records

Stage	Admissions	Patients	Events / note
AKI: baseline creatinine missing	3,869	—	
AKI: baseline creatinine available	6,537	6,198	
AKI: excluded by creatinine range	318	—	
AKI: preoperative RRT excluded next	24	—	
AKI: preoperative rise excluded next	68	—	
AKI: baseline eligible	6,127	5,832	
AKI: postoperative creatinine missing	368	—	Not classified as no AKI
AKI: final evaluable risk set	5,759	5,479	394 events
MI/stroke: final risk set	10,406	9,888	100 events
MI / stroke / both	—	—	15 / 86 / 1
MI/stroke and subsequent death	—	—	11

Exclusions are sequential and mutually exclusive within each endpoint. Patient counts are not subtracted across stages because patients may have repeated admissions. RRT, renal replacement therapy. ICU records are limited by the source release window. MI/stroke are newly recorded diagnoses under the locked algorithm, and death is not a component.

B. Postoperative creatinine observation among baseline-eligible admissions

Variable	Observed (n=5,759)	Unobserved (n=368)	SMD
Age, years	61.83	53.95	0.539
Anesthesia duration, min	240.07	144.08	0.801
Early iSHR	0.91	0.84	0.347
First-sample offset, min	29.23	24.20	0.360
Whole-anesthesia glucose count	2.78	1.70	0.592
Sex M	3,085 (53.6%)	65 (17.7%)	0.809
ASA 1.0	985 (17.1%)	122 (33.2%)	-0.376
ASA 2.0	3,668 (63.7%)	192 (52.2%)	0.235
ASA 3.0	948 (16.5%)	45 (12.2%)	0.121
ASA 4.0	58 (1.0%)	3 (0.8%)	0.020
ASA 5.0	4 (0.1%)	0 (0.0%)	0.037
ASA Missing	96 (1.7%)	6 (1.6%)	0.003

Variable	Observed (n=5,759)	Unobserved (n=368)	SMD
Emergency surgery 1	500 (8.7%)	34 (9.2%)	-0.020
Preoperative diabetes True	1,017 (17.7%)	68 (18.5%)	-0.021
Department GS	1,791 (31.1%)	31 (8.4%)	0.594
Department NS	1,164 (20.2%)	4 (1.1%)	0.652
Department OG	169 (2.9%)	258 (70.1%)	-1.947
Department OL	66 (1.1%)	12 (3.3%)	-0.144
Department OS	2,066 (35.9%)	15 (4.1%)	0.867
Department OT	4 (0.1%)	21 (5.7%)	-0.341
Department PS	16 (0.3%)	5 (1.4%)	-0.120
Department UR	483 (8.4%)	22 (6.0%)	0.093

Continuous entries are means; categorical entries are n (%). Signed standardized mean differences compare observed with unobserved admissions within the 6,127 baseline-eligible admissions, not with all early admissions. Observation differed substantially by department; this is a selection diagnostic, not a weighted outcome analysis. Missing ASA is shown separately.

Table S12. Early secondary-outcome model results and diagnostics

A. Model estimates and tests

Model	N/events	Exposure form	OR (95% CI)	Overall P	Nonlinear P
AKI, linear	5,759/394	Per 0.10	1.049 (0.995–1.104)	0.074	—
AKI, RCS	5,759/394	RCS, 2 df	—	0.021	0.046
MI/stroke, linear	10,406/100	Per 0.10	1.104 (1.033–1.179)	0.003	—
MI/stroke, RCS	10,406/100	RCS, 2 df	—	0.017	0.402
ICU, core	10,216/1,882	Per 0.10	1.139 (1.102–1.177)	<0.001	—
ICU, core + count	10,216/1,882	Per 0.10	1.130 (1.092–1.169)	<0.001	—

All models adjust for age, sex, ASA category and missingness, emergency status, diabetes, HbA1c, log2 anesthesia duration, surgical specialty, and first-sample timing. The ICU count-adjusted model additionally includes log2 whole-anesthesia glucose count. RCS knots are 0.69410913, 0.86776860 and 1.12151356, with patient-clustered HC1 intervals and tests. P values

are not adjusted for multiple comparisons. RCS results are reported alongside linear effect summaries.

B. Fit and influence diagnostics

Model	Parameters/ rank	Max leverage	Max Cook D	Max |DFBETA|	Flagged records
AKI, linear	14/14	0.055	0.0158	0.404	583
AKI, RCS	15/15	0.069	0.0151	0.411	582
MI/stroke, linear	14/14	0.127	0.0378	0.521	178
MI/stroke, RCS	15/15	0.181	0.0386	0.522	179
ICU, core	14/14	0.019	0.0046	0.211	2318
ICU, core + count	15/15	0.019	0.0048	0.216	2293

All six models converged with full design rank and finite coefficients; no GLM boundary warning occurred. Flags count records exceeding $2/\sqrt{N}$ for any standardized coefficient-change diagnostic; they are screening counts, not exclusions. No automatic individual deletion or new influence refit was performed. Apparent calibration intercepts/slopes near 0/1 describe in-sample fitting only and are not internal validation; the archived diagnostic data retain them.

C. Exposure support

Model	Observed range	Figure range (P5–P95)	In figure: N/events	At ≥1.50: N/events
AKI, RCS	0.354–2.575	0.636–1.263	5,193/333	108/14
MI/stroke, RCS	0.354–3.120	0.638–1.217	9,366/82	153/7

All eligible admissions enter each fit. The 1.50-versus-1.00 contrasts in Table 4 are within observed exposure values but above the 95th percentile; sparse high-value support should be considered. Figure S5 does not claim that the cropped display contains all fitted observations.

Table S13. Early first-glucose and eAG joint model

Joint exposure coefficients

Term	β per 20 mg/dL	Clustered SE	OR	95% CI	P
First early glucose	0.160	0.053	1.173	1.058–1.301	0.002
eAG	-0.173	0.088	0.841	0.708–0.999	0.049

Difference restriction

Restriction	Estimate	SE	Wald χ²	df	P	Interpretation
β_glucose + β_eAG = 0	-0.013	0.079	0.027	1	0.870	Difference restriction; not a ratio test

Standardized risks at selected glucose/eAG values

First glucose (mg/dL)	eAG (mg/dL)	Risk (%)	95% CI (%)
120	100	1.177	0.863–1.602
150	100	1.466	1.020–2.103
120	120	1.002	0.808–1.243
150	120	1.251	0.974–1.607
120	150	0.786	0.605–1.020
150	150	0.984	0.784–1.236

Same additive model for all panels: 10,406 admissions, 101 deaths, 9,888 patients; adjustment terms were age, sex, ASA, emergency status, diabetes, log2 anesthesia duration, department, first-60-minute sampling offset, and log2 total-anesthesia glucose count (age_decade; male; asa_high; asa_missing; emergency; diabetes; log2_duration; dept_OS; dept_NS; dept_Other; first60_offset_10min; log2_glucose_count); patient-clustered HC1 SE. Coefficients are per 20 mg/dL. No glucose-by-eAG interaction was fitted. The equal-and-opposite restriction is on untransformed glucose units and yields glucose minus eAG; it does not establish or test a glucose/eAG ratio. Risks are pointwise delta-method standardized predictions over the common EARLY_ALL covariate distribution; the selected display points are within the supported display range. The model is additive on the log-odds scale.

Table S14. Early monitoring frequency interaction

Early iSHR slope by whole-anesthesia glucose count

Whole-anesthesia glucose count	Admissions	Patients	Deaths	OR per 0.10 early iSHR	95% CI	P
1	2,971	2,903	23	1.060	0.908–1.239	0.459
2	3,456	3,400	23	1.182	1.071–1.304	0.001
≥3	3,979	3,878	55	1.114	1.023–1.213	0.013

Model diagnostics and joint interaction test

Metric	Value
Admissions	10,406
Patients	9,888
Deaths	101
Parameters / rank	18 / 18
Converged	TRUE
Boundary	FALSE
Joint interaction Wald χ^2 (df=2)	1.636 (P=0.441)
Maximum absolute score	8.890e-13
Condition number	648.951

All rows use the same early-iSHR interaction model (N=10,406 admissions, 101 deaths), adjusted for HbA1c, first-sample timing, and the core covariates; patient-clustered HC1 SE. The joint interaction test is reported below. The interaction is a 2-df joint test across the three whole-anesthesia glucose-count strata; no continuous log2 count term was added to this interaction model.

Table S15. Subsequent glucose rise conditional on the first measurement

Initial, conditional, and count-adjusted coefficients

Model	Term	β	Robust SE	OR	95% CI	P
Initial model	First glucose	0.183	0.057	1.201	1.074–1.343	0.001
Conditional model	First glucose	0.207	0.064	1.230	1.084–1.396	0.001
Conditional model	Subsequent rise	0.066	0.072	1.068	0.927–1.231	0.363
Conditional + count	First glucose	0.141	0.064	1.152	1.015–1.307	0.028
Conditional + count	Subsequent rise	-0.053	0.069	0.948	0.828–1.085	0.439

All three models use the same strict later-measurement cohort (7,435 admissions in 7,161 patients; 78 deaths) and the same clinical covariates, including HbA1c and first-sample timing. The initial model contains first glucose; the conditional model adds subsequent rise, and the final model further adds log2 whole-anesthesia glucose count. Glucose coefficients and ORs are per 20 mg/dL. The later rise is the maximum glucose after the first early glucose minus that first value, restricted to strictly later records; negative values are retained. These are conditional

associations, not causal or mediation estimates. The model without separate adjustment for first early glucose yielded an OR of 0.957 (0.810–1.132) per 20 mg/dL; its covariate set differs from the conditional models.

Table S16. Whole-anesthesia peak iSHR monitoring, sensitivity, and diabetes-interaction analyses

Analysis	N/events	Overall RCS P	Nonlinear P	OR(1.50 vs 1.00)	Linear OR (per 0.10)	Interaction P
Panel A Peak-iSHR core and sensitivity analyses						
Peak-iSHR core model	11,374/104	3.73e-05	0.456	1.83 (1.40–2.40)	1.13 (1.07–1.19)	—
First eligible admission per patient	10,782/90	3.06e-05	0.407	1.96 (1.45–2.67)	1.14 (1.07–1.22)	—
≥2 glucose measurements with measurement-frequency adjustment	7,948/79	0.021	0.317	1.46 (1.06–2.01)	1.08 (1.01–1.16)	—
Panel B Diabetes interaction analysis						
No diabetes	9,440/64	—	—	—	1.15 (1.08–1.22)	—
Diabetes	1,934/40	—	—	—	1.09 (0.999–1.19)	—
peak iSHR × diabetes interaction	11,374/104	—	—	—	—	0.310
Whole-anesthesia peak iSHR with measurement-count adjustment	11,374/104	—	—	—	1.09 (1.02–1.15)	—
Early iSHR primary-analysis reference (Table 2; Table S4)	10,406/101	5.97e-05	0.304	2.104 (1.481–2.989)	1.134 (1.063–1.210)	—
Panel C. Measurement-frequency interaction						
1 glucose measurement	3,426 / 25	—	—	—	1.070 (0.925–1.238)	—

Analysis	N/events	Overall RCS P	Nonlinear P	OR(1.50 vs 1.00)	Linear OR (per 0.10)	Interaction P
2 glucose measurements	3,678 / 23	—	—	—	1.128 (1.009–1.262)	—
≥3 glucose measurements	4,270 / 56	—	—	—	1.115 (1.041–1.194)	—
Joint peak iSHR × frequency test	11,374 / 104	—	—	—	—	0.842

The first-eligible-admission and repeated-measurement analyses used the whole-anesthesia peak-iSHR specification; the latter additionally adjusted for log2 measurement count. The full-cohort count-adjusted analysis used the whole-anesthesia peak-iSHR exposure. The primary first-60-minute iSHR analysis is summarized in Table S4. Diabetes and measurement-frequency interactions used linear peak iSHR. Frequency-specific ORs were estimated jointly in one interaction model (joint P=0.842).

Table S17. Whole-anesthesia peak iSHR and mortality under alternative adjustment sets

Model / analysis	N / deaths	df*	OR per 0.10 (95% CI)	P value
Panel A. Same-cohort whole-anesthesia peak-iSHR model comparison				
Peak-iSHR core covariates	11,374 / 104	12	1.128 (1.066–1.194)	3.41e-05
+ creatinine and malignancy	11,374 / 104	15	1.126 (1.063–1.193)	4.85e-05
+ creatinine, malignancy and low-MAP proportion	11,374 / 104	16	1.085 (1.020–1.153)	0.009
+ creatinine, malignancy and blood-product exposure	11,374 / 104	16	1.099 (1.038–1.165)	0.001
+ creatinine, malignancy, low-MAP proportion and blood-product exposure	11,374 / 104	17	1.064 (0.999–1.133)	0.052

Model / analysis	N / deaths	df*	OR per 0.10 (95% CI)	P value
Panel B. Measurement count added to the final Panel A model				
Final Panel A model + glucose count	11,374 / 104	18	1.045 (0.979–1.116)	0.182
Panel C. TWA models; common MAP subset				
Peak-iSHR core covariates, MAP subset	11,312 / 100	12	1.116 (1.054–1.183)	1.83e-04
+ measurement count	11,312 / 100	13	1.071 (1.007–1.138)	0.029
Expanded + count + TWA65	11,312 / 100	18	1.039 (0.973–1.109)	0.256
Expanded + count + TWA55	11,312 / 100	18	1.049 (0.983–1.119)	0.149
Expanded + count + TWA65, alternate MAP rule	11,312 / 100	18	1.043 (0.977–1.113)	0.210
Expanded + count + TWA55, alternate MAP rule	11,312 / 100	18	1.054 (0.989–1.123)	0.102

Panel D. Existing burden summary under the main MAP-processing rule

Measure in 11,312 eligible admissions	Median
Time below 65 mmHg, min	10.36
Time below 55 mmHg, min	0.00
TWA65 deficit, mmHg	0.169
TWA55 deficit, mmHg	0.000

These summaries use the main rule (finite MAP 20–200 mmHg; ≥80% connected-time coverage). They were read from the saved descriptive output; they are not event frequencies or additional regression models.

Panel A compares whole-anesthesia peak-iSHR models in the same 11,374 admissions. The model with core covariates, creatinine, and malignancy omits hypotension and blood-product terms if these are considered possible events after glycemic stress; the model adding both terms

treats them as proxies for shared operative severity. These models describe alternative conditional associations, not a formal mediation analysis. Creatinine was available in 7,133 admissions and median-imputed with a missingness indicator; malignancy was a recorded pre-anesthesia diagnosis. Hypotension was measured as the low-MAP reading proportion. Panel A omits measurement count; Panel B adds it. Panel C uses 11,312 MAP-eligible admissions with 100 deaths and replaces the low-MAP proportion with TWA65/TWA55, defined using valid connected observation time. Blood-product exposure is a positive-record indicator. Parameter counts exclude the intercept. In the peak-iSHR core RCS model, each doubling of anesthesia duration had an OR of 0.766 (95% CI 0.550–1.066).

Table S18. Whole-anesthesia peak glycemic expressions internal performance and coefficient restrictions

Panel A. Apparent and optimism-corrected performance

Model / metric	Apparent value	Apparent 95% CI	Optimism-corrected point estimate
Absolute peak glucose / C statistic	0.876578	0.844246–0.913389	0.865447
Absolute peak glucose / Brier	0.008587	0.007069–0.010023	0.008736
Absolute peak glucose / Calibration intercept	0.000000	Not reported: fitted-sample property	0.006586
Absolute peak glucose / Calibration slope	1.000000	Not reported: fitted-sample property	0.951113
Peak iSHR / C statistic	0.878490	0.846788–0.913882	0.867427
Peak iSHR / Brier	0.008581	0.007042–0.009995	0.008736
Peak iSHR / Calibration intercept	0.000000	Not reported: fitted-sample property	0.006864
Peak iSHR / Calibration slope	1.000000	Not reported: fitted-sample property	0.952223
Peak glucose + eAG / C statistic	0.879338	0.848314–0.914574	0.867345
Peak glucose + eAG / Brier	0.008590	0.007055–0.010020	0.008754
Peak glucose + eAG / Calibration intercept	0.000000	Not reported: fitted-sample property	0.006413
Peak glucose + eAG / Calibration slope	1.000000	Not reported: fitted-sample property	0.948274

Panel B. Paired differences from absolute peak glucose

Paired comparison / metric	Apparent difference	95% CI for apparent difference	Corrected point difference
Peak iSHR minus Absolute peak glucose / C_statistic	0.0019120	-0.0018229 to 0.0059136	0.0019801
Peak iSHR minus Absolute peak glucose / Brier	-6.164e-06	-0.0000976 to 0.0000345	2.291e-08
Peak glucose plus eAG minus Absolute peak glucose / C_statistic	0.0027592	-0.0001521 to 0.0086871	0.0018977
Peak glucose plus eAG minus Absolute peak glucose / Brier	3.252e-06	-0.0000846 to 0.0000315	0.0000180

Panel C. Glucose-difference restriction on the untransformed scale

Joint model	βG + βeAG (SE)	Wald χ^2 (df=1)	P value
Joint glucose/eAG model	-0.015048 (0.077165)	0.038030	0.845
joint with count	-0.020770 (0.078392)	0.070198	0.791

Panels A–B use 11,374 admissions, 10,782 patients and 104 deaths. All 1,000 paired patient-level bootstrap replicates were valid. The common comparison covariates omit the separate HbA1c term and add log2 count; the peak-iSHR association model still includes HbA1c. Apparent bootstrap percentile intervals do not apply to corrected differences. Apparent calibration intercepts near 0 and slopes near 1 are expected in the fitted sample. The glucose-plus-eAG model has 13 nonintercept parameters; the other two have 12. Fit criteria are retained in Table S18. Panel C tests coefficients for concentrations scaled per 20 mg/dL: $\beta G+\beta eAG=0$ represents a glucose-minus-eAG term, not a ratio. Its core and count-adjusted conditional models are distinct from the performance models. Code audit confirmed that each bootstrap model was evaluated in its resampled training data and the full analysis sample; correction is apparent minus mean training–test optimism. Near-degenerate training calibration intervals were removed rather than presented as validation precision.

Table S18 (continued). Whole-anesthesia peak-iSHR fit criteria

Model	Apparent C (95% CI)	Corrected C	AIC / BIC
Absolute peak glucose	0.8766 (0.8442–0.9134)	0.8654	945.50 / 1040.90
Peak iSHR	0.8785 (0.8468–0.9139)	0.8674	941.98 / 1037.39
Peak glucose + eAG	0.8793 (0.8483–0.9146)	0.8673	944.25 / 1046.99

These rows report the whole-anesthesia peak-iSHR exposure in its matched performance population; they are distinct from the first-60-minute iSHR models in the main Table 3.

Table S18 (continued). Whole-anesthesia peak-iSHR corrected error and calibration

Model	Corrected Brier score	Corrected calibration slope
Absolute peak glucose	0.008736	0.9511
Peak iSHR	0.008736	0.9522
Peak glucose + eAG	0.008754	0.9483

Whole-anesthesia peak-iSHR optimism-corrected points are reported. The model identities and coefficient-sum restriction remain separate from the first-60-minute iSHR comparison.

Supplementary Figures

Figure S1. Monitoring patterns and count-specific early-iSHR mortality associations

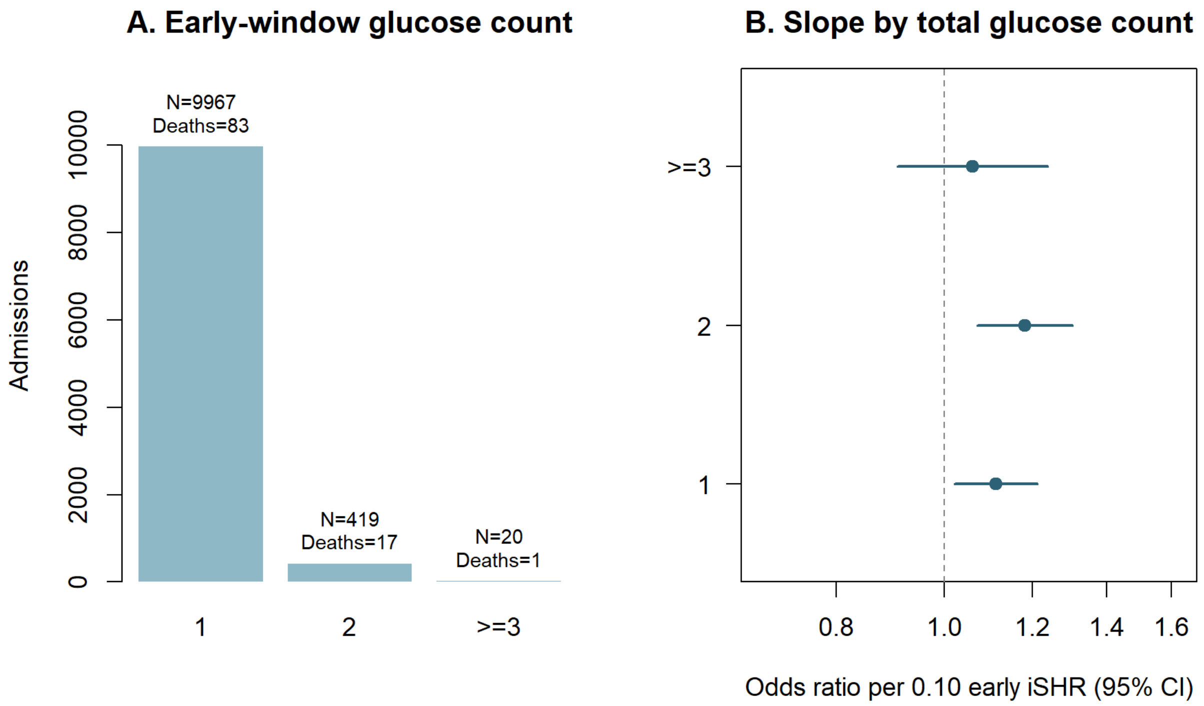

The figure uses EARLY_ALL (10,406 admissions; 101 deaths). Sampling summaries distinguish whole-anesthesia count from count within the early window. Count-specific linear ORs per 0.10 early iSHR come from one interaction model with core covariates, category main effects and two product terms; bars are 95% patient-clustered CIs. The joint interaction P value is 0.441. The whole-anesthesia groups contain 2,971/23, 3,456/23 and 3,979/55 admissions/deaths for 1, 2 and ≥3 measurements, respectively. Early-window counts are a different variable (9,967/419/20).

Figure S2. Early-cohort cumulative incidence of in-hospital death and live discharge

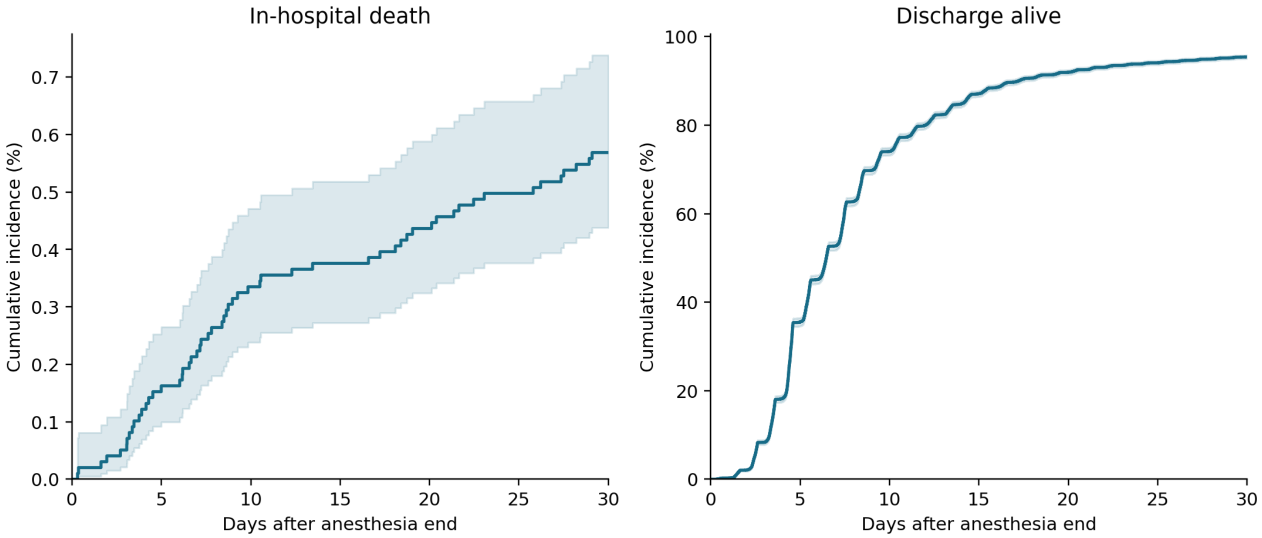

Aalen–Johansen estimates in 9,849 first-eligible-admission patients with early exposure data. At 30 days, 56 deaths and 9,396 live discharges had occurred; 397 remained in hospital. Panels use different vertical scales. Discharge is a competing event, not complete post-discharge mortality ascertainment (Table S6).

Figure S3. Apparent calibration of the three early-expression models

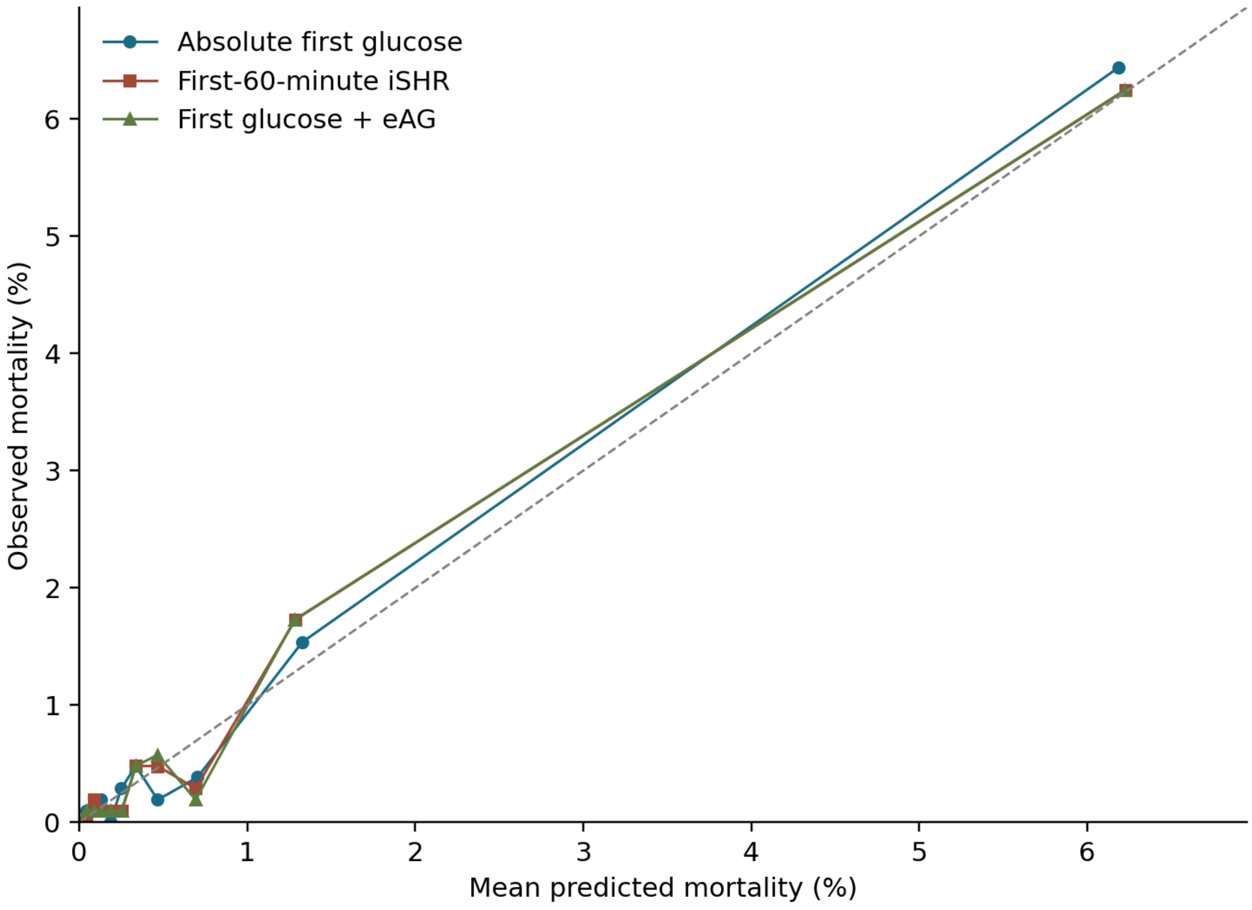

Observed event proportions and mean model predictions are grouped by fitted-risk decile in the 10,406-admission/101-death early cohort. This is apparent calibration, not a bootstrap-corrected calibration curve. Internal optimism-corrected metrics are separately reported in Table S7; no external validation was performed.

Figure S4. Apparent calibration of the core and expanded RCS models

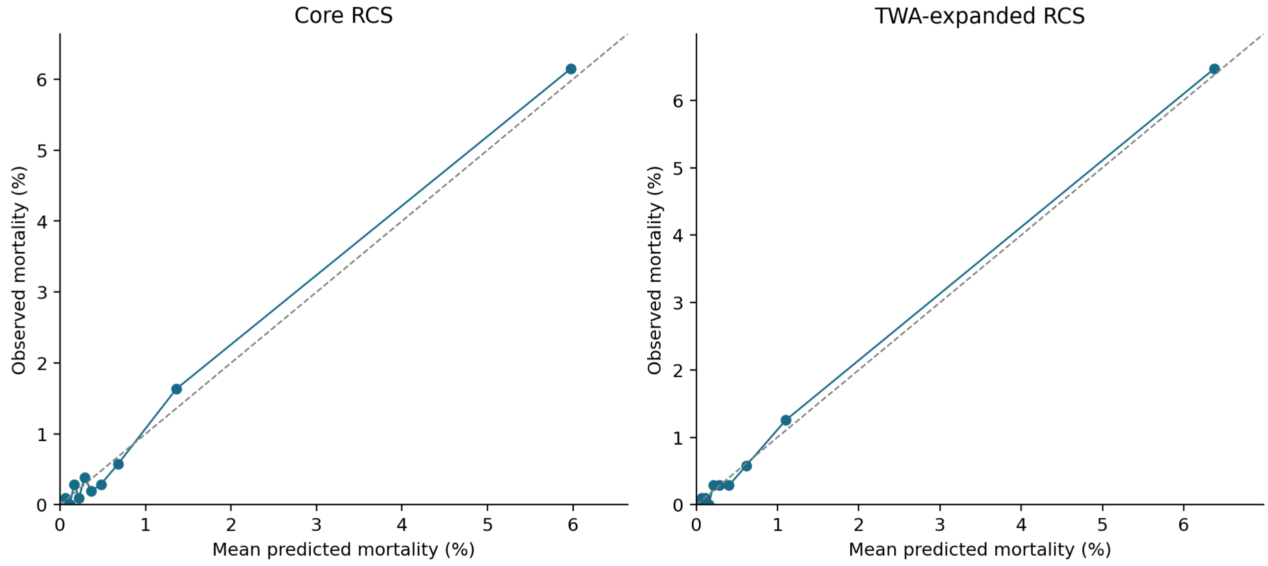

Core and TWA-expanded association models are shown separately in their eligible populations. Points are observed mortality and mean predictions in fitted-risk deciles; the diagonal indicates agreement. These plots are apparent checks, not proof of model validity. Their separate 500-resample corrected calibration estimates are in Table S9.

Figure S5. Early iSHR and secondary outcomes

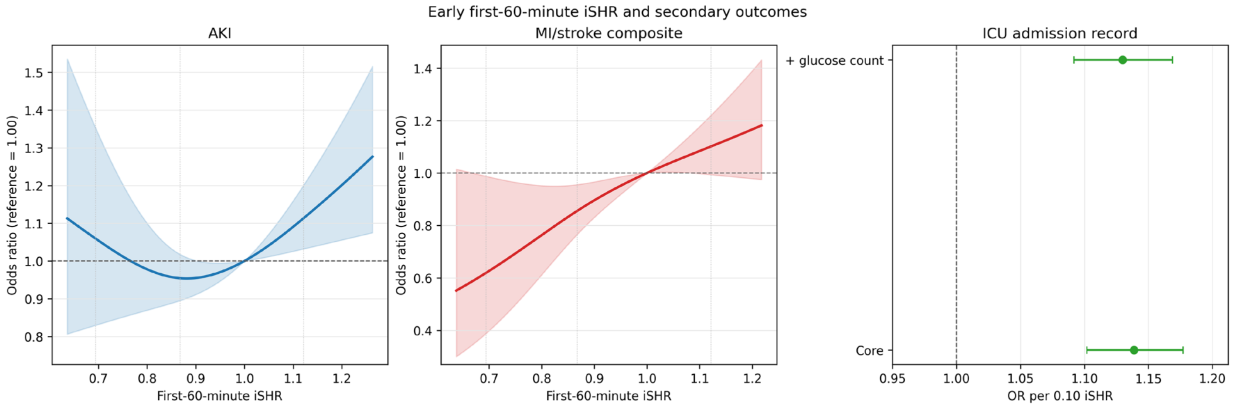

Exploratory outcome-specific models. ICU estimates are linear ORs per 0.10. AKI and MI/stroke curves use the frozen early knots (0.69410913, 0.86776860, 1.12151356), reference 1.00 and patient-clustered 95% CIs; no new knots were selected. Each spline panel displays its own observed 5th–95th percentile range, with full-cohort estimation. AKI: 5,759 admissions/394 events, 5,193 admissions/333 events within the plotted range; MI/stroke: 10,406/100, with 9,366/82 within range. The tabulated 1.50 contrast lies above the displayed 95th percentile but within observed exposure values; support at ≥1.50 is 108/14 for AKI and 153/7 for MI/stroke. Numerical results and diagnostics are in Tables S11, S12.

Figure S6. Whole-anesthesia peak-iSHR association curve and observed distribution

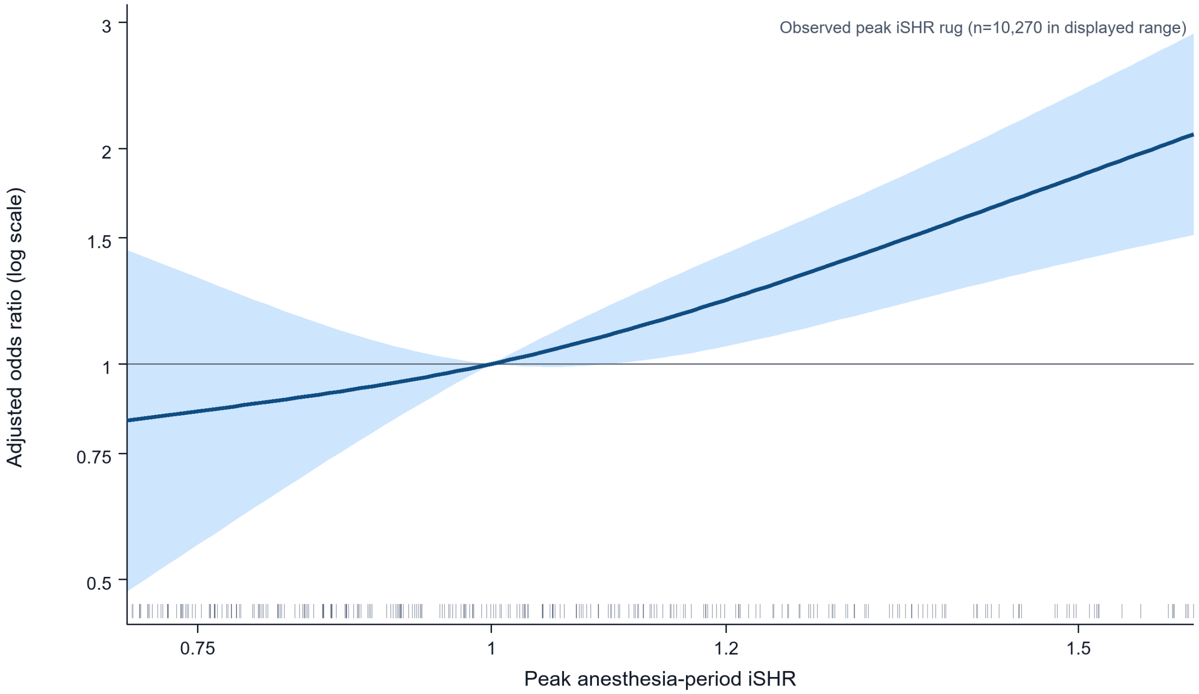

The solid line shows the whole-anesthesia peak-iSHR core-adjusted OR relative to 1.00; shading denotes the 95% CI. Short marks along the x-axis show observed whole-anesthesia peak-iSHR values. All 11,374 admissions (104 deaths) contributed to model fitting; 10,270 admissions lie within the displayed 5th–95th percentile range.
